# Facial representations of complex affective states combining pain and a negative emotion

**DOI:** 10.1038/s41598-024-62423-2

**Published:** 2024-05-22

**Authors:** Marie-Hélène Tessier, Jean-Philippe Mazet, Elliot Gagner, Audrey Marcoux, Philip L. Jackson

**Affiliations:** 1 School of Psychology, Université Laval, Québec City, Canada; 2 Centre for Interdisciplinary Research in Rehabilitation and Social Integration (Cirris), Québec City, Canada; 3 CERVO Brain Research Centre, Québec City, Canada; 4 Department of Computer Science and Software Engineering, Université Laval, Québec City, Canada

**Keywords:** Human behaviour, Computational science, Emotion

## Abstract

Pain is rarely communicated alone, as it is often accompanied by emotions such as anger or sadness. Communicating these affective states involves shared representations. However, how an individual conceptually represents these combined states must first be tested. The objective of this study was to measure the interaction between pain and negative emotions on two types of facial representations of these states, namely visual (i.e., interactive virtual agents; VAs) and sensorimotor (i.e., one's production of facial configurations). Twenty-eight participants (15 women) read short written scenarios involving only pain or a combined experience of pain and a negative emotion (anger, disgust, fear, or sadness). They produced facial configurations representing these experiences on the faces of the VAs and on their face (own production or imitation of VAs). The results suggest that affective states related to a direct threat to the body (i.e., anger, disgust, and pain) share a similar facial representation, while those that present no immediate danger (i.e., fear and sadness) differ. Although visual and sensorimotor representations of these states provide congruent affective information, they are differently influenced by factors associated with the communication cycle. These findings contribute to our understanding of pain communication in different affective contexts.

## Introduction

Imagine accidentally bumping your knee on a table while receiving a frustrating marketing phone call or watching disturbing war footage on television instead. These everyday situations reflect that pain is felt in various contexts and is rarely experienced alone. Indeed, everyday situations often involve other affective states, such as anger or sadness. Despite theoretical disagreements about the degree of overlap between the experience of pain and emotions^1^, it is acknowledged that these affective states share a negative valence (i.e., unpleasantness) and rely partly on mutual brain systems and regions, including the insula and anterior cingulate cortices (e.g., Ref.^2^). Experiencing other affective states can also modulate the pain experience itself. For example, concomitant negative emotions generally increase experienced pain whereas positive emotions reduce it^3^. Furthermore, the expression of pain and emotions often involves facial movements, some of which are common to both states^4^. However, pain is generally studied in isolation by experts, while research on emotions is not generally concerned with the pain state nor includes its communicative behaviors in experimental paradigms. These parallel research tracks have no doubt contributed to the sparsity of knowledge on how pain and emotional experiences interact at the representation and communication levels. To fill this gap, the current study focuses on the combined facial representations of pain and a negative emotion (anger, disgust, fear, or sadness) using recent technological tools (e.g., interactive virtual agents).

Pain is defined as “an unpleasant sensory and emotional experience associated with, or resembling that associated with, actual or potential tissue damage”^5^. It was historically linked to nociception and thus, a one-to-one relation with specific types of external stimulation (e.g., electric shocks, pressure, cold). Today, pain is characterized as a subjective experience involving multiple components including nociceptive, sensory, affective, and cognitive ones^6^. From an evolutionary perspective, pain can be communicated through facial configurations (i.e., patterns of visible contractions of facial muscles inferred as facial movements^7^) to trigger empathy and solicit help from others, or to promote survival by alerting others of danger^4^. Facial configurations can directly translate an inner state to prepare the body to respond adaptively to the situation or be aimed at others as communicative and social tools to influence others^8^. Current pain communication models distinguish the encoding process of experienced pain into behaviors from the decoding process of those behaviors by an observer to understand the other’ pain state^9^. The communication of the pain message is achieved through shared internal representations between an expresser and a perceiver of facial configurations associated with pain^10,11^ (also called conceptual knowledge or structure^12,13^). These representations are only accessible through communication outputs, thus driving both the encoding and decoding processes in an individual. These distinct communication processes are modulated by several individual factors related to the expression and perception of facial configurations (e.g., levels of empathy^14^, alexithymia^15^, and current mood^16^). Facial configurations are behaviors that are encoded and decoded by the same individual and could thus serve as a proxy for intraindividual variations in internal representations.

The communication of pain through facial movements has been actively studied in the last 50 years^17^, mostly described using the *Facial Action Coding System* (FACS^18^). This atheoretical system was first used in the research domain of emotions and fragments facial configurations in terms of Action Units (AUs), the smallest visually discriminative facial movements associated with muscular relaxation or contraction. Intensity ratings are attributed to each AU, from rest to maximal possible muscle contraction. According to the FACS manual, some AUs are combined due to their similar muscular bases. Specifically, AUs 25, and 26 are merged as they are related to mouth opening^18^. Similarly, AUs 6 and 7 are coupled due to their implication in orbit closure^19^. Closing of the eyelids (coded as AU 43 or AU 45 depending on the duration of closure, see A2 p. 39 in Ref.^18^) is frequently combined with AUs 6–7 and associated with the completion of the movement (i.e., tense/tight eye closures; p. 62 in Ref.^18^). AUs 9 and 10 (nose wrinkling and upper lip raising) involve different steps of *levator labii superioris* muscle contraction^19^. AUs most frequently associated with the communication of pain and that are expected to be expressed when experiencing the affective state (i.e., stereotypical facial configuration^7^) include (see Table 1): AU 4 (furrowing of the brows), AUs 6–7-43/45 (tightening of the orbital region muscles with eyes closing), AUs 9–10 (nose wrinkling and upper lip raising), and AUs 25–26 (opening of the mouth)^19,20^. These AUs have been proposed to encode different components of the pain experience. For instance, movements around the eyes (i.e., AUs 6–7-43/45) are associated with the intensity or the sensory component, and eyebrows and lower facial movements (AU 4 and AUs 9–10) are linked to the unpleasantness or the affective component^21^. However, results from recent studies suggest that human observers are attentionally biased towards the affective component when decoding others’ pain from facial configurations^22,23^. This predominance of the affective component in the communication of pain might be explained by the degree of overlap between pain and other affective states.

**Table 1:** AUs most associated with pain, anger, disgust, fear, and sadness in the literature.

Emotion	AU															
	1 Inner brow raiser	2 Outer brow raiser	4 Brow lowerer	5 Upper lid raiser	6 Cheek raiser	7 Lid tightener	9 Nose wrinkler	10 Upper lip raiser	12 Lip corner puller	15 Lip corner depressor	17 Chin raiser	20 Lip stretcher	23 Lip tightener	25 Lips part	26 Jaw drop	43/45 Eyes closed/Blink
Pain			**✕**		**✕**	**✕**	**✕**	**✕**	**○**			**○**		**✕**	**✕**	**✕**
Anger			**✕**	**✕**		**✕**		**○**			**○**		**✕**	**○**	**○**	
Disgust							**✕**	**✕**		**○**	**○**		**○**			
Fear	**✕**	**✕**	**✕**	**✕**								**○**		**✕**	**✕**	
Sadness	**✕**		**✕**		**○**					**✕**	**○**			**○**	**○**	

✕: AU which is reported to occur with prototype; ○: AU which is reported as likely to occur with prototype and/or major variant. From Refs.^4,20,25,34–36^.

In her pioneering review on the facial expression of pain, Williams^4^ exemplified the complex relationships between the communication of pain and negative emotions with the results of a few studies. In one of these studies, AUs extracted from photos of people experiencing pain in different contexts (e.g., accidents, medical interventions) were found to be shared with stereotypical facial configurations of negative emotions (i.e., anger, disgust, fear, and sadness), but the degree of overlap between the pattern of pain and those of negative emotions was found to be small^24^. This result highlights the specificity of pain in relation to negative emotions (also found in Ref.^25^) and the unlikelihood that the facial configuration of pain represents a blend of negative emotions. In a study conducted on people suffering from chronic jaw pain, participants undergoing a painful clinical assessment expressed one or more negative emotions on their faces in addition to pain^26^. These negative emotions expressed during pain could further inform observers about the patients’ pain. In another study, untrained observers assessed pain and perceived emotions in patients undergoing blood sampling^27^. An analysis of the AUs expressed by the patients combined with the observers' assessment showed that fewer expressions of joy and more expressions of anger, fear, and disgust were present. Furthermore, expressions of disgust, joy, fear, and sadness predicted the intensity of pain expressions. Anger and fear perceived by observers predicted their assessment of patients' pain. The results of the latter study demonstrated that the expression of specific emotions could predict the pain expressed by others. Despite the specificity of the stereotypical facial configuration of pain, the encoding of pain experiences and the decoding of pain contexts by observers are thus influenced by the presence of facial configurations of emotions.

Unlike pain, there are as many definitions of emotions as there are theories^28^. It can generally be defined as an affective reaction elicited by exteroceptive or interoceptive stimulation and can be considered as the interface between an organism and its environment^29^. Emotions have been classified into categories (e.g., basic emotions such as joy, sadness, and anger^30^) or dimensions (e.g., emotional valence and arousal^31^). Facial movements are a multiplex communication medium that may convey both classifications (e.g., AU 9 can reflect a negative valence as well as a state, such as disgust^32^). They can also communicate more than one category or dimension. The perception of one or more classifications from facial movements is associated with emotional granularity (i.e., an individual’s ability to feel or perceive an affective event according to many different categories^7^). High emotional granularity (e.g., distinguishing between feelings of frustration and irritation in the same event of anger) is more common than previously believed. For example, single affective states are less frequently self-reported than combined affective states of the same valence in everyday life^33^. Consequently, combined affective states can be expressed and perceived through facial movements.

Facial configurations associated with pain may differ according to the emotion simultaneously elicited, as suggested by a previous study combining various basic emotions. The study of Du, Tao, & Martinez (2014)^34^ aimed to describe compound facial configurations that were elicited by the description of situations in which the combination of more than one emotion may occur (e.g., angrily surprised is expressed when a person does something unexpectedly wrong to them). Their results demonstrated that facial movements commonly associated with basic emotions could be added into the same facial configuration and perceived in visually discriminable categories (e.g., angrily surprised = anger [AU 4] + surprise [AUs 25–26]). Yet, no study has investigated empirically combined affective states using other states than emotions, such as pain.

Table 1 shows AUs (as identified by Refs.^4,20,25,34–36^) associated with the stereotypical facial configuration of pain and negative emotions. The stereotypical facial configuration of pain shares facial movements with those of negative emotions, such as anger (AU 4), and disgust (AUs 9–10). However, it includes certain patterns of movements (AUs 6–7-43/45) that are considered to uniquely represent the experience of pain^22,25^. Likewise, the stereotypical facial configurations of negative emotions seem to have specific facial movements not shared with pain (e.g., AU 5 for fear and AU 15 for sadness^24,35^). Thus, facial configurations of pain combined with a negative emotion might vary according to the level of similarity between stereotypical representations of the two affective states.

Facial configurations can be categorized as spontaneous (i.e., triggered automatically by an underlying state or event), or posed (i.e., simulated or voluntarily displayed, but not necessarily felt). For example, spontaneous facial configurations of pain were studied in the context of people receiving a painful stimulus while patterns of facial movements were being recorded (e.g., Ref.^37^). Posed facial configurations were rather studied in the context where pain was depicted on the faces of non-expert models’ or actors’ faces in the absence of a painful stimulus (e.g., Ref.^25^). Spontaneous and posed facial configurations generally share the same facial movements but show varying temporal patterns in the encoding stage of pain communication^38^. Spontaneous facial configurations reflect the congruent display of a felt affective state, whereas posed facial configurations depict the simulation of an affective state. From a communication perspective, spontaneous facial configurations are perceived as the “genuine” ones. However, the study of posed facial configurations promotes a better understanding of how people internalize the learned social display norms to facially express affective states (e.g., Ref.^39^). Thus, posed facial configurations give insight into conscious stereotypical representations of facial configurations that support both encoding and decoding processes.

The decoding of affective states through faces encompasses different mechanisms leading to the perception of an affective state^40^. These mechanisms include distinct information (e.g., seeing the picture of an expressing face) that triggers internal representations (e.g., mental simulation of the expressing face) and may lead to an output (e.g., motor imitation). While classical models of face processing have primarily focused on visual mechanisms (e.g., Ref.^41^), current models also include sensorimotor and conceptual mechanisms to analyze facial configurations of emotions^42^. These mechanisms could interact with each other. For example, disrupting individuals’ facial movements impairs their capacity to distinguish facially expressed emotions from perceptually similar distractors^43^. However, visual, conceptual, and sensorimotor mechanisms do not always provide congruent emotional information about the perceived facial configuration^44^. Recent findings have shown that individuals affected by congenital facial paralysis can still recognize emotions from facial configurations, even without a sensorimotor representation^45^. Visual, sensorimotor, and conceptual representations of facial configurations emerge from partially independent mechanisms to perceive affective states.

An individual’s internal representations of facial configurations are difficult to access explicitly and can only be measured through expressive or perceptual behaviors. Recent progress in digital technologies offers new ways to study the behaviors associated with these representations. Several automatic recognition algorithms that promote measuring the experience of pain are now available^46^. However, few of them focus on differentiating pain from negative emotions. Other computer-vision algorithms, such as OpenFace^47^, were developed to detect facial movements indiscriminately from the affective states portrayed. They have the advantage of being less time-consuming and less burdensome than manual coding of the facial movements by FACS^48^. OpenFace was also shown to be as accurate (i.e., F1 scores of > 90%) as expert human coders when images of facial configurations are captured in laboratory settings^49^. It is both a fast and precise tool to investigate the encoding of affective states through the production of facial configurations.

Interactive virtual agents (i.e., human characters digitally represented and integrated into a user-friendly interface, VAs) can be used to create dynamic and realistic stimuli of facial configurations to study participants’ perceptions (e.g., Ref.^50^). The advantage of VAs in the study of facial configurations includes the control of each AU and its intensity (i.e., high internal validity) while representing a certain photographic and behavioral realism with the human face (i.e., moderate generalization)^50^. The individual control of AU provides unique information on the specific role of each facial movement and its interaction with other movements, leading to the generation of facial configuration^51^. They can also serve as a customizable visual template for participants to create a wide range of facial configurations directly from their own mental representation, for example, via web-based applications (e.g., the E-Smiles-Creator^52^, “thisemotiondoesnotexist”^53^, genetic algorithms^54^). With VAs, participants do not have to voluntarily produce certain facial movements with difficulty^55^ or be limited by the number of examples of facial configurations (by video or image stimuli) that can be presented during an experiment. For example, VAs were used to generate the participants’ preferred facial configurations of happiness, fear, sadness, and anger in a recent study^56^. The results of this study reveal that the representations of the preferred configurations vary between individuals, with significant overlap between fear and sadness facial configurations. These individual differences affect the participants’ recognition of emotions (i.e., the decoding process): the more similar the test stimuli were to their internal representation of the emotion, the more participants recognized them as manifestations of that emotion category^57^. Although innovative and well-constructed, the current applications were not developed to represent combined affective states beyond basic emotions and allow changes in individual facial movements. Therefore, the development of VAs with customizable AUs can provide new insights into how people mentally depict facial configurations of combined affective states.

The study of combined affective states has so far focused on a few emotions. However, it should be broadened to include other important and well-defined states such as pain. Indeed, pain and emotions share several characteristics and are interrelated in certain disorders (e.g., chronic pain^58^). In addition, the processes of encoding and decoding combined affective states via facial configurations have mainly been studied in isolation and not with the same individuals. The use of new technological tools (i.e., automatic recognition algorithm and interactive VAs) now makes it possible to investigate the different facial representations of combined affective states in the same study. A better understanding of these representations in communication processes is an essential first step towards investigating how the combined state of pain and negative emotions is expressed and perceived.

The main purpose of this study (Obj. 1) was to examine how four negative emotions (anger, disgust, fear, and sadness) interact with pain on visual (i.e., VAs) and sensorimotor (i.e., one’s production) representations of these combined states. On one hand, it was hypothesized that the intensity of facial movements shared with stereotypical representations of negative emotions (i.e., anger and disgust) and pain^20,25^ would increase on VAs and the faces of the participants. On the other hand, the intensity of these movements would decrease for negative emotions that have stereotypical representations distinct from that of pain (i.e., sadness and fear). A secondary objective (Obj. 2) was to assess whether visual (i.e., imitating a model) and conceptual (i.e., from one’s internal representation) information about the facial configurations provide a congruent sensorimotor representation of the combined affective states. It was hypothesized that the production of facial movements would be distinct between the imitated and posed facial configurations, considering that some facial movements, such as inner brow raising (AU 1), are more difficult to voluntarily imitate without an underlying affective context than other facial movements, such as furrowing of the brows (AU 4)^55^.

## Methods

### Participants

The sample size of the study consisted of 28 healthy participants (15 women) aged between 18 and 40 years old (mean age = 26.25 ± 6.11 years). To determine the final sample, a power analysis based on 1000 Monte-Carlo simulations was done on the preliminary data of the first seven participants who completed the *Virtual Agents* task (see *Material and Measures*). The analysis targeted the effect of the affective state (corresponding to the five types of scenarios) in a linear mixed-effects model conducted on the intensity of AU 4. The *simr* v. 1.05 and *lmerTest* v. 3.1–3 packages of *R* (v. 4.2.2) were used in *Rstudio* v 2022.07.2^59,60^. The results showed that a sample between 25 (80.40%, 95% CI [77.80–82.82]) and 30 (86.20%, 95% CI [83.91–88.28]) participants was necessary to reach a power of 80%.

Participants were excluded if they reported having a neurological or psychiatric disorder or a pain condition, worked with people suffering from a pain condition (e.g., health workers who are exposed frequently to pain expressions), or had previously participated in a study on pain expressions from our research laboratory. Participants were recruited by emails sent to *Université Laval*’s students and employees lists, and posters displayed in the community of the Quebec City area. The study was approved by *Centre intégré universitaire de santé et de services sociaux de la Capitale-Nationale*’s Ethics Committee (#2020–1824). All participants voluntarily gave their written informed consent for their participation and received a compensation of 25 Canadian dollars. The research was conducted in accordance with the relevant guidelines and regulations (e.g., Declaration of Helsinki).

### Materials and measures

**Figure 1:**
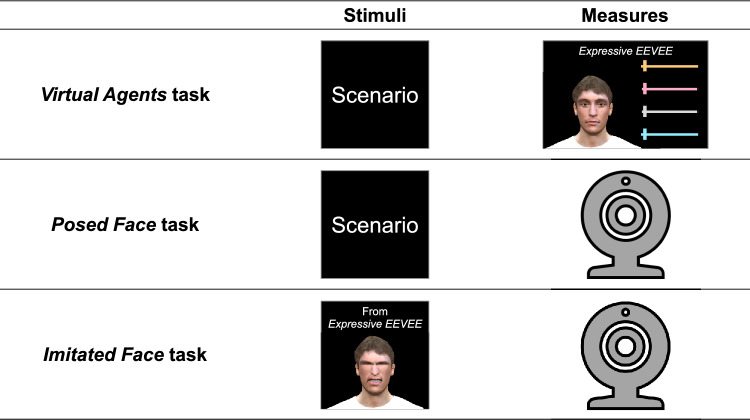
Stimuli and measures of the three computer tasks: Virtual Agents, Posed Face, and Imitated Face. Participants were asked to represent the facial configuration most likely expressed by characters in the scenarios on the virtual agents' faces (Virtual Agents task) and on their own faces (Posed Face task). They also had to imitate the facial configurations previously created on the virtual agents (Imitated Face task). The facial movements (i.e., AUs) were extracted from the Expressive EEVEE application (Virtual Agents task) and photographs of the participants’ faces (Posed Face and Imitated Face tasks).

The study was divided into three computer tasks (Fig. 1). All participants took part in these tasks where they were asked to represent the facial configuration most likely expressed by characters in scenarios involving combined affective states of pain and a negative emotion on VAs’ faces (*Virtual Agents* task) and on their own faces (*Posed Face* task). Participants also had to imitate the facial configurations previously created on the VAs (*Imitated Face* task). A photograph of the participant’s face was taken to extract the facial configuration representing the character’s affective state in the written scenario (*Posed Face* task) or that of the VA (*Imitated Face* task). These tasks were followed by subjective control measures. They all ran under Psychopy v. 3.2.4^61,62^. The main computer monitor was an Acer GN246HL (1920 × 1080 px resolution, 60.96 cm display size, 60 Hz refresh rate) on top of which a Logitech C922 Pro Stream webcam (720p resolution) was placed for *Posed Face* and *Imitated Face* tasks.

#### Creation of scenarios

Participants were presented with scenarios (i.e., short texts) describing fictional characters in different daily situations involving pain and a negative emotion. They had to imagine the affective state of pain combined with a negative emotion that the character could express in the presented context. Five conditions of scenarios were created. Pain was combined with a negative emotion according to four experimental conditions: anger (Anger-Pain), disgust (Disgust-Pain), fear (Fear-Pain), or sadness (Sadness-Pain). A control condition describes individuals in different daily situations involving strictly pain (Pain).

Fifteen scenarios (three for each condition; see Supplementary Table S1 online) composed of three simple sentences were previously validated through an online study (see Supplementary Information Appendix 2 online). Each scenario began with a sentence describing the situation’s context (Context), followed by a sentence implying an action leading to the painful situation and experience (PainExp). A sentence eliciting a negative emotion was added before or after pain (Emotion), evenly distributed among the 15 scenarios (8 with the order Emotion-Context-PainExp, and 7 with the order Context-PainExp-Emotion). For the three control scenarios involving only pain (Pain), the sentence implying a negative emotion was substituted by additional details describing the individual’s pain experience (e.g., sensory characteristics).

#### Virtual agents task

##### Creation of animated virtual agents

Two VAs, representing a young male and a young female, were selected from the platform *EEVEE* (Empathy-Enhancing Virtual Evolving Environment; for more details, see Ref.^50^). They were created by selecting 3D models and skin textures from photos of white adults and were previously validated in an online study (see Supplementary Information Appendix 3 online for a summary of data validating their age, gender, and perceived realism, and see Ref.^63^ for full details of the validation study). The faces of the VAs were animated using blend shapes which are pre-programmed linear changes of the 3D model forming a mesh. Intensity value ranging from 0 to 100%, with 1% increments, was linked to each blend shape and determined the range of motion of the mesh within the 3D environment. The associations between AUs and blend shapes, and their intensities (minimal to maximal intensity: 0 to 100%) were determined by a developer with a FACS Coder certification (based on FACS manual^18^). Twelve AUs or clusters of AUs were depicted on the VAs based on the stereotypical facial configurations of pain, anger, disgust, fear, and sadness (see Table 1): AU 1, AU 2, AU 4, AU 5, AUs 6–7-43/45, AUs 9–10, AU 12, AU 15, AU 17, AU 20, AU 23, and AUs 25–26. The combinations of AUs to communicate negative emotions and pain were validated in a perceiver-dependent way in a series of experiments^50^. For each VA, the same animation of blend shapes, thus the same AUs magnitudes, was applied using Blender v. 2.79b software (Blender Foundation).

##### Expressive EEVEE

Both VAs were imported into the Unreal Engine v. 4.19 game-development platform (Epic Games Inc.) to create an interactive application called *Expressive EEVEE*. This application allowed the design of facial configurations on the VAs (see Supplementary Information Appendix 4 online). The *Expressive EEVEE* interface consisted of two sections on a screen. On the left, one of the VAs was presented and, on the right, the sliders were displayed to control the facial movements of the VA. The presentation between the male and female VA was randomized among trials for each participant.

In order to create a user-friendly interface, nine sliders were used to manipulate the 12 AUs (or clusters of AUs), moving symmetrically on both sides of the VA’s face (see Supplementary Table S4 online). They were presented as continuous two- or three-marker scales. Two-marker scales ranged from the minimum (0% at the left end) to the maximum intensity of the facial movement(s) (100% at the right end). Three-marker scales combined AUs on the same slider and ranged from the minimum (0% in the middle) to the maximum intensity of the facial movement(s) (− 100% at the left end and 100% at the right end). AUs with opposing movements^18^ were on the same slider to allow participants to create humanly possible facial configurations. AU 16 and AU 24 were added to oppose AU 12 and AU 20 respectively but were not included in the analyses considering they could not be detected on the participants’ faces by the automatic recognition algorithm used in this study (see *Preprocessing and Planned Analyses*). Also, AUs frequently combined in the context of the affective states of interest^4,20,25,34–36^ were assigned to different sliders. The nine sliders were divided into two tabs to reduce the visual load on the screen: four upper-face sliders and five lower-face sliders. The participants could navigate between the tabs as needed.

In the *Virtual Agents* task, a second computer monitor (Dell UP Compag LA2205wg with 1680 × 1050 px resolution, 55.9 cm display size, and 60 Hz refresh rate) was simultaneously used to display the scenarios, instructions, and other measures. It was placed at a 160° angle to the right of the main monitor displaying the *Expressive EEVEE* interface. The participants were required to represent the facial configuration most likely expressed by the character in the scenario on the VA. Final values on the sliders were recorded and included in the analyses. A picture of the resulting facial configuration on the VA was also collected to be used for the *Imitated Face* task.

#### Posed face task

Participants were required to pose the facial configuration most likely expressed by the character in the scenario. They could practice beforehand their facial configuration without visual feedback (i.e., no mirror). Once ready, participants had to press the Enter key on the keyboard to take a still frame of their face. The resulting photos were included in the analyses.

#### Imitated face task

The participants were asked to imitate the facial configuration previously created on the VA in the *Virtual Agent* task. As in the *Posed Face* task, they practiced their facial configuration without visual feedback of their face and, when they were ready, they pressed the Enter key on the keyboard to take a still frame of their face. The resulting photos were included in the analyses.

#### Subjective control measures and questionnaires

Two subjective control measures followed the tasks. The recognition of each single affective state (i.e., anger, disgust, fear, sadness, or pain) was measured to validate participants’ perception of distinct affective states in the scenarios. After the *Virtual Agents* and *Posed Face* tasks, the participants indicated the affective state(s) they had perceived from the character in the scenario among five options (i.e., pain, anger, sadness, fear, and disgust). Each option was assigned to a specific key on the keyboard. One or more affective states could be selected. The final selection was validated by pressing the spacebar. The level of confidence was used to measure the participants’ meta-cognition and decision-making abilities^64–66^. Participants were asked to rate their level of confidence in the facial representations made in all tasks (i.e., *Virtual Agents*, *Posed Face*, and *Imitated Face*). They used a continuous three-marker scale, from left to right: “Not at all confident”, “Uncertain”, and “Totally confident” (as in Ref.^66^). Keyboard arrows were used to select the level of confidence that needed to be confirmed by pressing the spacebar.

Three validated and standardized questionnaires and one homemade questionnaire were administered to quantify the characteristics of participants relevant for decoding affective states and the use of VA. All questionnaires were converted into an online format using the Dexero FD v. 6.5.5 web platform (Dexero Inc.).

The French-Canadian version of the Positive and Negative Affect Schedule (PANAS) questionnaire^67,68^ was used to measure the momentary affective state of the participants before initiating the computer tasks (e.g., “To what extent you feel this way right now, that is, at the present moment?”). This self-reported measure includes two lists of ten positive (PA) and negative (NA) affective state adjectives which are rated on a 5-point Likert scale ranging from “Very slightly or not at all” to “Extremely”. Individual scores from the two subscales (sum of PA and NA items) were calculated.

The French version of the Interpersonal Reactivity Index (IRI) questionnaire^69,70^ was used to measure participants’ self-reported empathy, defined as the capacity to share and understand another’s feelings without confusing them with our own^71^. For each of the 28 items of the IRI, the participant indicated the extent to which the item corresponded to them on a 5-point Likert scale ranging from “Does not describe me well” to “Describes me very well”. A total score, as well as four individual scores from the Fantasy (F), Empathic Concern (EC), Personal Distress (PD), and Perspective Taking (PT) subscales were calculated.

The French version of the Toronto Alexithymia Scale (TAS-20) questionnaire^72,73^ was used to measure participants’ self-reported alexithymia, defined as the interindividual differences in one’s ability to distinguish and communicate experienced emotions^74^. This trait is characterized by difficulties in identifying and expressing emotions, an impoverished fantasy life, difficulty in distinguishing feelings from bodily sensations, and thoughts essentially oriented towards concrete concerns. The TAS-20 consists of 20 items rated on a 5-point Likert scale ranging from “Strongly disagree” to “Strongly agree”. A total score and three individual scores from the Difficulty Identifying Feelings (DIF), Difficulty Describing Feelings (DFD), and Externally Oriented Thinking (EOT) subscales were calculated.

The ExpVA questionnaire was developed to collect socio-demographic information (e.g., age, sex, gender, native language) and to document the participants’ level of experience with virtual characters (e.g., in video games and animated television series/movies).

### Procedure

Participants took part in one laboratory session lasting approximately 150 min. They sat approximately 60 cm in front of the main computer monitor on top of which the webcam was placed. The height of the chair was adjusted to align the participant’s face to the center of the video frame. To optimize the quality of the photos, participants were asked to uncover their forehead to fully show their eyebrows and to wear contacts for those who needed corrective lenses. In addition, a 60-W lamp located behind the main computer monitor illuminated the participant’s face without dazzling them.

After completing the consent forms, the PANAS was administered to the participants. The computer tasks were then explained. The order of the *Virtual Agents*, *Posed Face*, and *Imitated Face* tasks was counterbalanced in three variations so that *Virtual Agents* always preceded *Imitated Face*. The remaining questionnaires (IRI, TAS-20, and ExpVA) were administered after the computer tasks. Participants’ comments and feedback about the study were also collected.

In the *Virtual Agents* task, a trial started with a fixation cross for 2 s, followed by the presentation of a scenario. The trial duration started when participants moved any slider to change the VA’s facial characteristics. Time was indicated on a clock at the top right of the screen. Unlimited time was given to the participants, but the clock turned orange to indicate that 60 s had passed and they had to move on to the next trial to keep within the time available for the study. Once satisfied with the facial configuration created, the participants stopped the time by clicking on the clock marking the end of the trial. The subjective measures (level of confidence and affective state(s) perceived) were then recorded. This 45-min task was preceded by four practice trials and consisted of 30 pseudo-randomized trials corresponding to 15 scenarios per VA (a male and a female).

The course of a trial in the practice and experimental sessions was mostly the same in the *Posed Face* and *Imitated Face* tasks. A trial began with a 2-s fixation cross, followed by the presentation of one of the scenarios or VA pictures, practice time, and a photograph of the participant’s face. At the end of the trial, both subjective measures (level of confidence and affective state(s) perceived) were recorded in the *Posed Face* task, while only the level of confidence was collected in the *Imitated Face* task. Four practice trials preceded the experimental trials. The *Posed Face* task consisted of 30 pseudo-randomized trials corresponding to 15 scenarios, each repeated two times, for a total duration of about 20 min. The *Imitated Face* task consisted of 30 pseudo-randomized trials corresponding to 15 scenarios represented on the two VAs (a male and a female) in the *Virtual Agents* task, for a total duration of about 10 min.

### Preprocessing and planned analyses

The data from Psychopy, Unreal, and Dexero platforms were extracted and included in a common database. The photos of the participants’ faces (from *Posed Face* and *Imitated Face* tasks) were processed using OpenFace v. 2.2.0^47^. This software is a toolkit that implements automatic facial behavior analyses, including AUs recognition, from image, video, or webcam outputs. For each frame, the algorithm detects facial landmarks to estimate the presence and intensity of AUs. The AUs classification model of OpenFace was trained on a range of datasets comprising videos of people responding to affective-elicitation tasks, for instance, patients with shoulder pain performing a series of range-of-motion as found in the UNBC-McMaster Shoulder Pain Expression Archive Database^75^. Moreover, it was found as a reliable tool in a previous study for detecting AUs in photos of posed facial configurations of pain taken in a controlled laboratory setting (average of pain-related AUs: recall = 90.1%, precision = 73.7% and accuracy = 72.6%^76^). In the current study, 12 AUs (or clusters of AUs) were targeted: AU 1, AU 2, AU 4, AU 5, AUs 6–7-43/45, AUs 9–10, AU 12, AU 15, AU 17, AU 20, AU 23, and AUs 25–26. For each trial, the estimated presence and intensity of AUs from the participants’ final performed facial configuration were considered in the analyses.

Data-driven (i.e., machine learning) and hypothesis-driven (i.e., inferential statistics) analyses were carried out to compare the affective states depicted on the VAs and the participants’ faces (as part of Obj. 1) and to discriminate between imitated and posed facial configurations (as part of Obj. 2). Both types of analyses are complementary in their approach to the present problem^77^. On the one hand, machine learning considers multivariate non-linear trends that can be assumed from many complex variables (e.g., 12 AUs × 3 tasks to detect different combined affective states). It can reveal unsuspected differences or relationships between the variables. Machine learning models can also be used as a proof-of-concept of new ideas and an empirical validation of the obtained results. On the other hand, inferential statistics test hypotheses from theoretical backgrounds (i.e., psychological theories on how affective states are combined). The interpretability of the results on the variables studied is thus maximized. This procedure of combining analyses was previously used in other studies with similar questions and data^78,79^.

#### Machine learning

Through a supervised machine learning approach, classification models were conducted to compare affective states on the intensity of the 12 AUs (or clusters of AUs) on the VAs and the participants’ faces. The same procedure was also chosen to discriminate imitated and posed facial configurations. Using a classification model to demonstrate that most of the different facial configurations of combined states are discriminable from one another based on AUs is a method that was previously used (e.g., Ref.^34^) and confirms that the average facial configuration emerging from each affective state results in distinct facial configurations. The versions of the Python libraries used to conduct the machine learning experiments are listed in Supplementary Information Appendix 5 (online).

Pre-analysis and visualization of the data suggested complex and subtle relationships between AUs to distinguish affective states and types of facial configurations (see Supplementary Figs. S10, S12, and S14 online). Based on that observation, a Multilayer Perceptron (MLP) machine learning model with one hidden layer was selected. The data, grouped by participant ID, was shuffled and partitioned into a tuning dataset (6 participants, 21.43%) for optimizing the models’ hyperparameters, and a training and testing dataset (22 participants, 78.57%) for evaluating the models’ performance. A five-fold cross-validation procedure was conducted on the tuning dataset to estimate the best hyperparameters. Subsequently, the tuned models were trained and evaluated via a ten-fold cross-validation procedure, repeated ten times, on the rest of the data. The mean of the 100 composite test accuracy scores was chosen as the main performance metric of the classification models. Based on a game theory approach^80^, the SHapley Additive exPlanation values (SHAP^81^) for each AU (or clusters of AUs) were computed to illustrate the relative importance of AUs in distinguishing affective states or imitated and posed facial configurations, and to further interpret the models’ operations. This technique was used successfully in a previous study to reflect the relative importance of AUs in detecting moderate pain from innocuous leg pressure^82^. The AUs identified as the most important according to SHAP were included in the following inferential statistics analyses. The following criteria were applied for inclusion of the most (and least) relevant AUs: (1) top- (and lowest-) ranked feature according to the absolute mean of SHAP; (2) feature(s) statistically indistinguishable from top- (and lowest-) ranked feature.

#### Inferential statistics

Analyses of statistical inferences were performed with SPSS Statistics for Macintosh v. 25 and Windows v. 29 (IBM Corp.). For all analyses, the alpha level was set at a standard level of 0.05. When necessary, simple effects and post hoc pairwise comparisons were tested using Bonferroni-corrected levels of significance (ɑ_simple_ _effects_ = 0.15/7 = 0.214, and ɑ_post hoc_ = 0.05/10 = 0.005). The *p*-values already corrected by the Bonferroni adjustment are indicated with the subscript “bonf”. Only the statistically significant comparisons are reported in the *Results* section. Other comparisons not mentioned were thus found non-statistically significant (*p* ≥ 0.005, *p*_bonf_ ≥ 0.05).

Linear mixed-effects models (LMMs) were selected for the statistical inference analyses as a flexible alternative to repeated-measures analyses of variance (rANOVA; see Refs.^83,84^ for a description of the advantages of this type of analysis). A compound symmetry structure was used for the within-group correlation structure of all models. LMMs were conducted separately for the intensity of the AUs shared with pain and other relatively important AUs according to SHAP to compare the facial configurations depicted on the VAs between affective states. Two within-subjects variables were included as fixed effects factors: *Affective states* (5 conditions: Anger-Pain, Disgust-Pain, Fear-Pain, Sadness-Pain, and Pain) and *Gender of virtual agent* (2 conditions: Female and Male). Gender was included as a control variable, as previous research has found that the perception of pain differs between male and female VAs^85^. *Gender of virtual agent* is not mentioned in the *Results* section, as no interaction with *Affective states* was found for all AUs. Moreover, LMMs were conducted separately for the intensity of the AUs shared with pain and other relatively important AUs according to SHAP to compare imitated and posed facial configurations of participants according to affective states. Two within-subjects variables were included as fixed effects factors: *Type of facial configurations* (2 conditions: Imitated and Posed) and *Affective states* (5 conditions: Anger-Pain, Disgust-Pain, Fear-Pain, Sadness-Pain, and Pain).

## Results

For each participant and condition (five affective states from two facial representations, as well as, where applicable, two genders of VA and two types of facial configurations), AUs intensities were averaged. Due to technical problems, part of one participant’s data was excluded from the analyses (i.e., 1.8% of all AUs data in the *Imitated Face* task and 1.4% of all AUs data in the *Virtual Agents* task). Graphs illustrating the results were created using Python libraries: Matplotlib v. 3.7.2^86^, Seaborn v. 0.12.2^87^, and Plotly v. 5.9.0 (Plotly Technologies Inc.).

## Subjective control measures and questionnaires

Table 2 presents the socio-demographic information and mean scores or frequencies on questionnaires, as well as the results of subjective control measures. The mean levels of the PANAS subscales matched published student norms (positive affective states = 39.7 ± 7.9, and negative affective states = 14.8 ± 5.4)^67^. Similarly, mean IRI and TAS-20 total scores were consistent with previous student samples (respectively, IRI total score = 67.22 ± 9.20^85^, and TAS-20 total score = 47.39 ± 10.37^88^). In addition, the overall mean accuracy of recognizing the affective states from the scenarios was 93.62 ± 5.37%. Miss rates were higher for recognizing anger (18.27%), disgust (23.66%), and fear (22.98%) compared to sadness (11.01%) and pain (3.87%). More false alarms were found for only recognizing pain (10.98%) compared to other single affective states (anger = 2.83%, disgust = 0.83%, fear = 1.88%, and sadness = 3.26%), which is unsurprising given the presence of pain in all scenarios. Furthermore, the descriptive statistics showed the same moderate level of confidence for *Imitated Face*, *Posed Face*, and *Virtual Agents* tasks (67.88% to 69.75%). Only one participant in the *Virtual Agents* and the *Imitated Face* tasks had a confidence level below 50% (respectively 47.24% and 48.36%), and two participants in the *Posed Face task* (48.06% and 49.98%), corresponding to levels ranging from ‘not at all confident’ to ‘uncertain’. This moderate level of confidence in producing complex affective states has already been observed in a previous study^89^.

**Table 2:** Participants' frequencies and mean responses to the questionnaires and subjective control measures.

Variables	
Sample (n)	28
Age [18–40 years old]	26.25 (6.11)
Main cultural affiliation	
French Canadian	19 (67.86%)
Others	9 (32.14%)
Number of years of education [12–26 years]	18.50 (2.77)
Highest level of education achieved	
High school or CÉGEP^1^ degree	6 (21.43%)
Undergraduate degree	13 (46.43%)
Master and doctorate degree	9 (32.14%)
Principal occupation	
Student	24 (85.71%)
Worker	4 (14.29%)
ExpVA	
Do you play or did you ever play video games?	
Yes	25 (89.29%)
In the last month, how many hours per week did you play video games? [0–15 h]	3.13 (4.59)
No	3 (10.71%)
Do you watch or did you ever watch animated series or movies?	
Yes	25 (89.29%)
In the last month, how many animated series or movies did you watch [0–20 animated series or movies]	3.44 (4.56)
No	3 (10.71%)
PANAS	
Positive affect [16–43]	31.75 (6.33)
Negative affect [10–18]	12.11 (2.41)
IRI	
Perspective taking [6–27]	19.46 (4.91)
Fantasy [5–27]	18.82 (5.26)
Empathic Concern [10–27]	19.96 (3.82)
Personal Distress [0–18]	10.21 (4.92)
Total [46–87]	68.46 (10.86)
TAS-20	
Difficulty describing feelings [5–23]	14.18 (5.23)
Difficulty identifying feelings [7–29]	16.14 (5.41)
Externally-oriented thinking [12–23]	16.25 (3.12)
Total [30–65]	46.57 (10.29)
Recognition of each affective state	
Accuracy of anger recognition [83.33–100.00]	94.08 (4.96)
Accuracy of disgust recognition [78.33–100.00]	94.63 (6.13)
Accuracy of fear recognition [85.00–100.00]	93.90 (5.22)
Accuracy of sadness recognition [61.44–100.00]	95.16 (8.06)
Accuracy of pain recognition [61.67–100.00]	90.32 (8.30)
Mean accuracy of single affective state [78.00–100.00]	93.62 (5.37)
Levels of confidence	
Virtual agents task [47.24–99.18]	69.75 (12.59)
Posed face task [48.06–98.98]	69.29 (12.69)
Imitated face task [48.36–98.58]	67.88 (12.49)
Mean level of confidence [49.47–98.92]	68.97 (11.87)

Numbers in brackets represent the range of the variable, and numbers in parentheses represent the standard deviation for the mean responses or the proportion in % of participants.

*ExpVA:* Questions about video games and animated television series/movies, *PANAS:* Positive and Negative Affect Schedule, *IRI:* Interpersonal Reactivity Index, *TAS-20:* 20-item Toronto Alexithymia Scale.

^1^i.e., college: first two or three years of post-secondary education in the province of Québec (Canada).

## Differences between affective states on virtual agents

### Relative importance of AUs on virtual agents

As part of Obj. 1, the MLP model predicted the affective states on the VAs with an accuracy of 46.43 ± 6.00% at the end of the cross-validation procedure, which was 26.43% more than a model predicting classes at random (i.e., 20%). Although weak (but consistent with other studies of non-verbal behaviors^79^), this accuracy suggests that the machine learning process acquired some knowledge from the relation between the features (i.e., AUs). The class with the best accuracy was Sadness-Pain (70%) and the one with the least accuracy was Disgust-Pain (21%; see Supplementary Fig. S11 online). Examples of facial configurations on the virtual agents best classified by the machine learning model are presented in Supplementary Information Appendix 6 (online).

**Figure 2:**
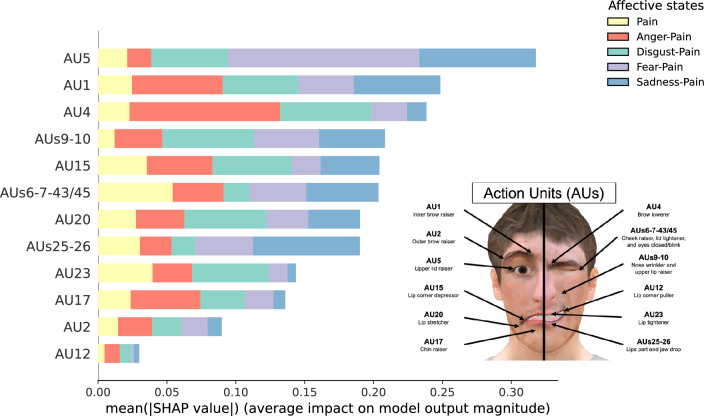
Absolute mean of SHAP indicating the relative importance of the intensity of the 12 AUs (or clusters of AUs) depicted on the virtual agents to predict affective states.

Figure 2 shows the absolute mean of the SHAP computed from the MLP model for the intensity of the AUs depicted on the VAs according to the affective state. The MLP model mainly relied on AUs 1, 4, 5, 6–7-43/45, and 15 while rarely relying on AU 12 (see Supplementary Fig. S17 online). The model predominantly relied on AU 4 to predict Anger-Pain (mean SHAP = 0.109), AUs 9–10 to predict Disgust-Pain (mean SHAP = 0.067), AU 5 to predict both Fear-Pain (mean SHAP = 0.139) and Sadness-Pain (mean SHAP = 0.084), and AUs 6–7-43/45 to predict Pain (mean SHAP = 0.054).

### AUs shared with pain and other important AUs on virtual agents

**Figure 3:**
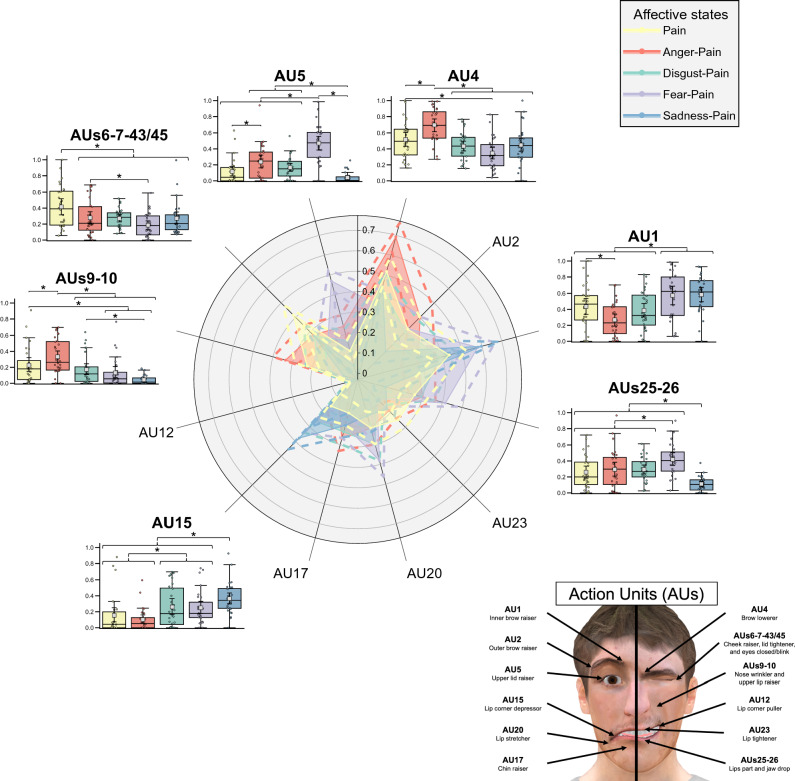
Results of the Affective states effect on the mean intensity of AUs depicted on the virtual agents. Colored dotted lines in the radar plot indicate a 95% CI for each affective state. The AUs with significant differences between affective states are shown beside the radar plot. The colored box-and-whisker plots and the colored points show the data distribution for each affective state (*n* = 28). The whiskers present the minimum and maximum values, the vertical length of the box presents the interquartile range, and the horizontal line within the box presents the median. The grey squares show the mean scores for each affective state and error bars indicate a 95% CI (Bootstrap = 1000) calculated by Seaborn. * *p*s_bonf_ < 0.05.

Figure 3 illustrates differences between affective states on the intensity of the 12 AUs (or clusters of AUs) depicted on the VAs. In line with the Obj. 1, the results regarding AUs shared with pain (i.e., AU 4, AUs 6–7-43/45, AUs 9–10, and AUs 25–26) and other relatively important AUs according to the SHAP (i.e., AU 1, AU 5, and AU 15) are described in the following paragraphs. The familywise inflation of Type I error rate from the multiple LMMs was controlled for by the Bonferroni adjustment (ɑ_univariate_ = 0.05/7 = 0.007).

The LMM on the intensity of AU 4 showed a statistically significant main effect of *Affective states* (*F*(4, 239.40) = 24.55, *p* < 0.001, η^2^_p_ = 0.29). The affective state associated with the highest AU 4 intensity was Anger-Pain. (*p*s_bonf_ < 0.001). Pain was more intense than Fear-Pain (*p*_bonf_ < 0.001). The LMM on the intensity of AUs 6–7-43/45 showed a statistically significant main effect of *Affective states* (*F*(4, 239.06) = 13.34, *p* < 0.001, η^2^_p_ = 0.18). The affective state associated with the highest AUs 6–7-43/45 intensity was Pain (*p*s_bonf_ < 0.001). Anger-Pain was more intense than Fear-Pain (*p*_bonf_ = 0.029). The LMM on the intensity of AUs 9–10 showed a statistically significant main effect of *Affective states* (*F*(4, 239.06) = 24.98, *p* < 0.001, η^2^_p_ = 0.30). The affective state associated with the significantly highest AUs 9–10 intensity was Anger-Pain (*p*s_bonf_ ≤ 0.005). Pain was more intense than Fear-Pain (*p*_bonf_ = 0.014) and Sadness-Pain (*p*_bonf_ < 0.001) Also, Disgust-Pain was more intense than Sadness-Pain (*p*_bonf_ < 0.001). The LMM on the intensity of AUs 25–26 showed a statistically significant main effect of *Affective states* (*F*(4, 239.26 = 23.88, *p* < 0.001, η^2^_p_ = 0.29). On one hand, the affective state associated with the highest AUs 25–26 intensity was Fear-Pain (*p*s_bonf_ ≤ 0.003). On the other hand, Sadness-Pain was the affective state associated with the lowest AUs 25–26 intensity (*p*s_bonf_ < 0.001).

The LMM on the intensity of AU 1 showed a statistically significant main effect of *Affective states* (*F*(4, 239.19) = 20.95, *p* < 0.001, η^2^_p_ = 0.26). The two affective states associated with the highest AU 1 intensity was Fear-Pain (*p*s_bonf_ ≤ 0.006) and Sadness-Pain (*p*s_bonf_ ≤ 0.001). Pain was more intense than Anger-Pain (*p*_bonf_ = 0.002). The LMM on the intensity of AU 5 showed a statistically significant main effect of *Affective states* (*F*(4, 239.24) = 45.26, *p* < 0.001, η^2^_p_ = 0.43). The affective state associated with the highest AU 5 intensity was Fear-Pain (*p*s_bonf_ < 0.001). Anger-Pain and Disgust-Pain were more intense than Sadness-Pain (*p*_bonf_ < 0.001 and *p*_bonf_ = 0.003). Also, Anger-Pain was more intense than Pain (*p*_bonf_ = 0.003). The LMM on the intensity of AU 15 showed a statistically significant main effect of *Affective states* (*F*(4, 238.91) = 26.12, *p* < 0.001, η^2^_p_ = 0.30). The affective state associated with the highest AU 15 intensity was Sadness-Pain (*p*s_bonf_ ≤ 0.001). Fear-Pain and Disgust-Pain were more intense than Pain (*p*_bonf_ = 0.004 and *p*_bonf_ = 0.003) and Anger-Pain (*p*s_bonf_ ≤ 0.001).

## Differences between affective states on participants’ posed and imitated faces

### Relative importance of AUs on participants’ faces

As part of Obj. 1, the MLP model predicted the affective states on the participants’ faces with an accuracy of 33.25 ± 3.72% at the end of the cross-validation procedure, which was 13.25% more than a model predicting classes at random (i.e., 20%). Although weak (but consistent with other studies of non-verbal behaviors^79^), this accuracy suggests that the machine learning process acquired some knowledge from the relation between the features (i.e., AUs). The class with the best accuracy was Fear-Pain (41%) and the one with the least accuracy was Anger-Pain (23%; see Supplementary Fig. S13 online).

**Figure 4:**
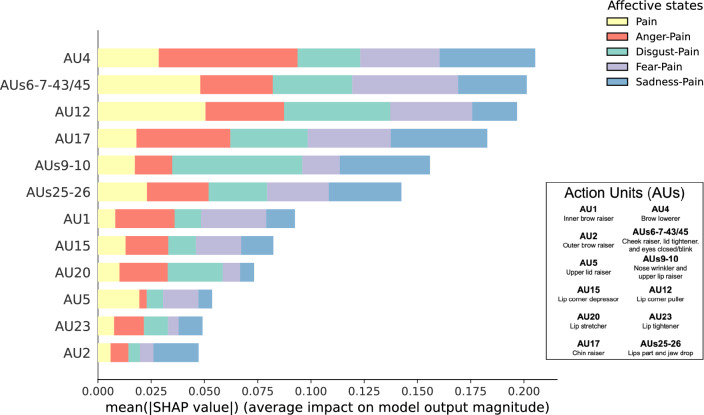
Absolute mean of SHAP indicating the relative importance of the intensity of the 12 AUs (or clusters of AUs) measured on the participants’ faces to predict affective states.

Figure 4 shows the absolute mean of the SHAP computed from the MLP model for the intensity of the AUs measured on the participants’ faces according to affective state. The MLP model mainly relied on AUs 4, 6–7-43/45, 12, and 17 while rarely relying on AUs 2, 5, and 23 (see Supplementary Fig. S18 online). The model predominantly relied on AU 4 to predict Anger-Pain (mean SHAP = 0.065), on AUs 9–10 to predict Disgust-Pain (mean SHAP = 0.061), on AUs 6–7-43/45 to predict Fear-Pain (mean SHAP = 0.050), on AU 17 to predict Sadness-Pain (mean SHAP = 0.045), and AU 12 to predict Pain (mean SHAP = 0.051).

As part of Obj. 2, another MLP model predicted the type of facial configurations (i.e., imitated or posed) on the participants’ faces with an accuracy of 63.62 ± 6.28% at the end of the cross-validation procedure, which was 11.25% more than a baseline model predicting the dominant class in the data (i.e., 52.37%). Although weak (but consistent with other studies of non-verbal behaviors^79^), this accuracy again implies that the machine learning process acquired some knowledge from the relation between the features (i.e., AUs). The two classes had a similar accuracy (62% for imitated and 63% for posed; see Supplementary Fig. S15 online).

**Figure 5:**
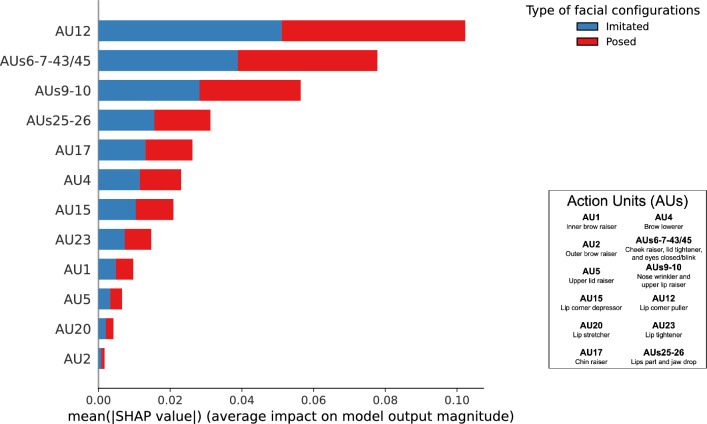
Absolute mean of SHAP indicating the relative importance of the intensity of the 12 AUs (or clusters of AUs) measured on the participants’ faces to predict the type of facial configurations.

Figure 5 shows the absolute mean of the SHAP computed from the MLP model for the intensity of the AUs measured on the participants’ faces according to the type of facial configurations. The MLP model mainly relied on AU 12 while rarely relying on AU 2 (see Supplementary Fig. S19 online). The importance of an AU (or a cluster of AUs) in the model was balanced between imitated and posed facial configurations (e.g., for imitated and posed AU 12, mean SHAP = 0.051).

### AUs shared with pain and other important AUs on participants’ faces

**Figure 6:**
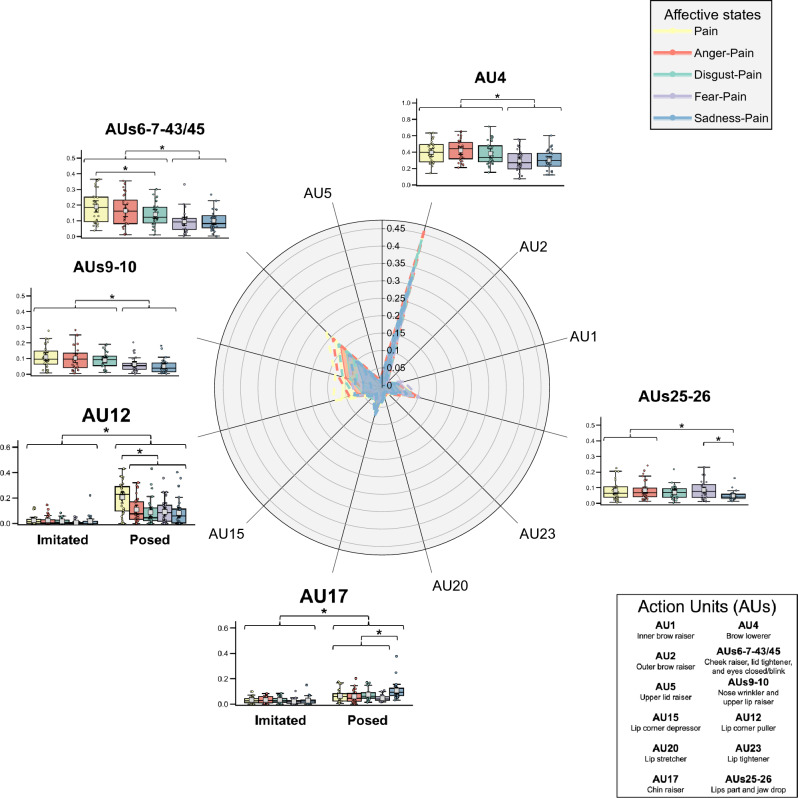
Results of the Affective states effect and the interaction effect with Type of facial configurations on the mean intensity of AUs measured on participants’ faces. Colored dotted lines in the radar plot indicate a 95% CI for each affective state. The AUs with significant differences between affective states are shown beside the radar plot. The colored box-and-whisker plots and the colored points show the data distribution for each affective state on imitated (*n* = 27) and posed (*n* = 28) facial configurations. The whiskers present the minimum and maximum values, the vertical length of the box presents the interquartile range, and the horizontal line within the box presents the median. The grey squares show the mean scores for each affective state on imitated and posed facial configurations, and error bars indicate a 95% CI (Bootstrap = 1000) calculated by Seaborn. *ps_bonf_ < 0.05.

Figure 6 illustrates the differences between affective states on the intensity of the 12 AUs (or clusters of AUs) measured on the participants’ imitated and posed faces. In line with the Obj. 1 and the Obj. 2, the results regarding AUs shared with pain (i.e., AU 4, AUs 6–7-43/45, AUs 9–10, and AUs 25–26) and other relatively important AUs according to the SHAP (i.e., AU 12 and AU 17) are described in the following paragraphs. The familywise inflation of Type I error rate from the multiple LMMs was controlled for by the Bonferroni adjustment (ɑ_univariate_ = 0.05/6 = 0.008).

The LMM on the intensity of AU 4 showed two statistically significant main effects: *Affective states* (*F*(4, 237.88) = 16.03, *p* < 0.001, η^2^_p_ = 0.21) and *Type of facial configurations* (*F*(1, 239.07) = 20.62, *p* < 0.001, η^2^_p_ = 0.08). No interaction effect was found statistically significant (*F*(4, 237.88) = 1.42, *p* = 0.228, η^2^_p_ = 0.02). AU 4 was more intensely represented on posed than imitated facial configurations. Anger-Pain, Disgust-Pain, and Pain were more intense than Fear-Pain (*p*s_bonf_ < 0.001) and Sadness-Pain (*p*s_bonf_ ≤ 0.001). Likewise, the LMM on the intensity of AUs 6–7-43/45 showed two statistically significant main effects: *Affective states* (*F*(4, 237.83) = 25.28, *p* < 0.001, η^2^_p_ = 0.30) and *Type of facial configurations* (*F*(1, 238.86) = 102.05, *p* < 0.001, η^2^_p_ = 0.30). No interaction effect was found statistically significant (*F*(4, 237.83) = 1.15, *p* = 0.336, η^2^_p_ = 0.02). AUs 6–7-43/45 were more intensely represented on posed than imitated facial configurations. Anger-Pain, Disgust-Pain, and Pain were more intense than Fear-Pain (*p*s_bonf_ < 0.001) and Sadness-Pain (*p*s_bonf_ ≤ 0.003). Also, Pain was more intense than Disgust-Pain (*p*_bonf_ < 0.001). The LMM on the intensity of AUs 9–10 showed two statistically significant main effects: *Affective states* (*F*(4, 238.09) = 18.29, *p* < 0.001, η^2^_p_ = 0.24) and *Type of facial configurations* (*F*(1, 239.12) = 63.44, *p* < 0.001, η^2^_p_ = 0.21). No interaction effect was found statistically significant (*F*(4, 238.09) = 1.51, *p* = 0.201, η^2^_p_ = 0.03). AUs 9–10 were more intensely represented on posed than imitated facial configurations. Anger-Pain, Disgust-Pain, and Pain were more intense than Fear-Pain (*p*s_bonf_ ≤ 0.026) and Sadness-Pain (*p*s_bonf_ < 0.001). Furthermore, the LMM on the intensity of AUs 25–26 showed two statistically significant main effects: *Affective states* (*F*(4, 237.71) = 5.37, *p* < 0.001, η^2^_p_ = 0.08) and *Type of facial configurations* (*F*(1, 239.35) = 92.64, *p* < 0.001, η^2^_p_ = 0.28). No interaction effect was found statistically significant (*F*(4, 237.71) = 2.11, *p* = 0.080, η^2^_p_ = 0.03). AUs 25–26 were more intensely represented on posed than imitated facial configurations. Sadness-Pain was less intense than Pain (*p*_bonf_ = 0.005), Anger-Pain (*p*_bonf_ = 0.002), and Fear-Pain (*p*_bonf_ = 0.001).

The LMM on the intensity of AU 12 showed a statistically significant interaction effect of *Affective states* X *Type of facial configurations* (*F*(4, 238.11) = 8.10, *p* < 0.001, η^2^_p_ = 0.12). AU 12 was more intensely represented on posed than imitated facial configurations for all scenarios (*p*s < 0.001). In posed facial configurations (*F*(4, 238.11) = 18.96, *p* < 0.001, η^2^_p_ = 0.24), the affective state associated with the highest AU 12 intensity was Pain (*p*s < 0.001). In imitated facial configurations, the comparison of affective states was not statistically significant (*F*(4, 238.11) = 0.32, *p* = 0.867, η^2^_p_ = 0.01). The LMM on the intensity of AU 17 also showed a statistically significant interaction effect of *Affective states* X *Type of facial configurations* (*F*(4, 238.27) = 4.22, *p* = 0.003, η^2^_p_ = 0.07). AU 17 was more intensely represented on posed than imitated facial configurations for all scenarios (*p*s ≤ 0.021). In posed facial configurations (*F*(4, 238.27) = 9.47, *p* < 0.001, η^2^_p_ = 0.137), the affective state associated with the highest AU 17 intensity was Sadness-Pain (*p*s ≤ 0.002). In imitated facial configurations, the comparison of affective states was not statistically significant (*F*(4, 238.27) = 0.39, *p* = 0.814, η^2^_p_ = 0.01).

## Exploratory analysis: level of information about pain in both representations

As an a posteriori analysis of Obj. 1, a pain index was calculated to globally measure the potential level of pain information expressed on the VAs and the participants’ faces for each affective state. It stems from the Prkachin and Solomon pain intensity (PSPI) metric, which defines pain on a frame-by-frame basis using the FACS^75^. The pain index is described as the sum or average of intensities of four AUs (or clusters of AUs) associated with most of the pain information (i.e., AU 4, AUs 6–7-43/45, AUs 9–10, and AUs 25–26^20^), resulting in a scale ranging from 0 to 1. A LMM was conducted for the pain index to compare the level of pain expressed on the VAs and the participants’ faces according to affective states. Two within-subjects variables were included as fixed effects factors: *Affective states* (5 conditions: Anger-Pain, Disgust-Pain, Fear-Pain, Sadness-Pain, and Pain) and *Type of facial representation* (2 conditions: VAs and participants’ faces).

**Figure 7:**
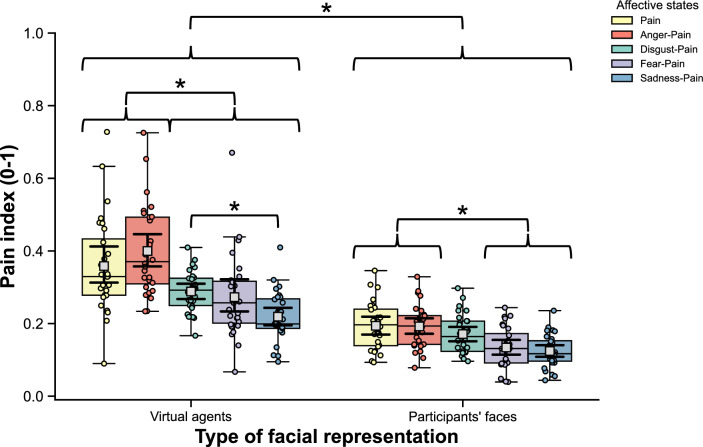
Results of the interaction effect of Affective states and Type of facial representation on the mean intensity of pain index. The colored box-and-whisker plots and the colored points show the data distribution for each affective state on the virtual agents and the participants’ faces (*n* = 28). The whiskers present the minimum and maximum values, the vertical length of the box presents the interquartile range, and the horizontal line within the box presents the median. The grey squares show the mean scores for each affective state on the virtual agents and the participants’ faces, and error bars indicate a 95% CI (Bootstrap = 1000) calculated by Seaborn. * *p*s_bonf_ < 0.05.

The LMM on the pain index showed a statistically significant interaction effect of *Affective state* X *Type of facial representation* (*F*(4, 241.98) = 5.64, *p* < 0.001, η^2^_p_ = 0.09). The pain index was higher on the VAs than participants’ faces for all affective states (*p*s < 0.001). On the VAs (*F*(4, 242.02) = 30.37, *p* < 0.001, η^2^_p_ = 0.33) and the participants’ faces (*F*(4, 241.95) = 6.19, *p* < 0.001, η^2^_p_ = 0.09), Pain and Anger-Pain were higher than Fear-Pain (*p*s ≤ 0.002) and Sadness-Pain (*p*s < 0.001). Additionally, on the VAs, Disgust-Pain was lower than Pain as well as Anger-Pain (*p*s < 0.001) and higher than Sadness-Pain (*p* < 0.001). Figure 7 presents the results on the pain index.

As a further exploratory step towards examining the facial configurations of combined states, the affective information expressed on virtual agents and participants’ faces was compared for each affective state (Pain, Anger-Pain, Disgust-Pain, Fear-Pain, and Sadness-Pain). An index for each negative emotion combined with pain (Anger, Disgust, Fear, and Sadness) was calculated. As with the pain index, these indexes aim to synthesize the information on anger, disgust, fear, and sadness conveyed by the facial configurations. Each index was computed as the average of a selection of AUs that are typically associated with negative emotions (see Table 1) and that were found important in the SHAP analyses either on VAs or participants’ faces. It results in a scale ranging from 0 to 1. Details about the results are found in Supplementary Information Appendix 8 (online).

## Discussion

To contribute to a deeper understanding of pain communication, other affective states need to be explored simultaneously. Pain is rarely experienced out of context and other affective states are prone to interact with its communication. Therefore, this study aimed to examine the combination of pain and four negative emotions (anger, disgust, fear, and sadness) on visual (i.e., VAs) and sensorimotor (i.e., one’s production) representations of facial configurations (Obj. 1). As predicted, fear and sadness decreased the intensity of the facial movements associated with pain (i.e., AUs 4, 6–7-43/45, 9–10, and 25–26) as displayed on the VAs and the participants’ faces. However, anger and disgust did not systematically amplify the intensity of these facial movements. Instead, the effect of anger was limited to specific facial movements on the VAs that are shared with the affective component of pain and certain negative emotions. This study also aimed to assess the congruence of sensorimotor representations of combined affective states based on visual (i.e., imitating a model) and conceptual (i.e., from one’s internal representation) information about the facial configurations (Obj. 2). The pattern of facial movements did not generally differ between imitated and posed facial configurations. Still, the intensity of facial movements was generally higher for posed than imitated facial configurations. These findings reveal the complex interaction between pain and emotions in facial representations.

The results of this study show that fear and sadness have a distinctive effect on the facial configuration typically perceived as pain compared to anger and disgust. In line with the decrease of facial movements associated with pain, facial movements not common to pain but typically associated with those two target negative emotions were more intensely represented in Fear-Pain (i.e., AUs 1, 5) and Sadness-Pain (i.e., AUs 1, 15, 17) scenarios compared to other scenarios. As identified by several authors^4,20,25,34–36^, some facial movements are shared between pain and certain negative emotions, such as anger (e.g., AU 7) and disgust (e.g., AUs 9–10), but other movements are emotion-specific, as for fear (e.g., AU 2) and sadness (e.g., AU 15). These results align with the analyses of AUs in compound facial configurations of basic emotions found in Du et al.^34^ and other studies (e.g., Refs.^12,13,56,57,90^) that contrast fear and sadness with anger, and disgust. The theory of the sensory modulation function of facial configurations^91–93^ might explain this finding. In this theory, the facial configurations associated with disgust and anger (and presumably pain) are opposed to those of fear based on antagonist action tendencies to augment or diminish sensory exposure. For instance, the upper lid raising (i.e., AU 5), frequently associated with the context of fear, could help the visual perception of the environment to detect a potential threat. On the contrary, the nose wrinkling (i.e., AU 9), frequently associated with the context of disgust (and pain) could block the odor intake of a threatening substance. From an evolutionary perspective, facial movements associated with certain affective contexts could thus originate as a sensory interface with the physical world in which at least two context-dependant patterns of behaviors are promoted: sensory vigilance and sensory rejection. Although the presumed physiological function of the stereotypical facial configuration of sadness remains unclear^8^, empirical findings suggest that the facial movements expected in a sad context differ from those anticipated in a disgusting or angry context^94^. Throughout evolution, the main function of affective states has shifted from the physiological regulation in response to environmental events (i.e., informing about internal states) to the social communication resulting from ritualization (i.e., exaggerating nonverbal behaviors to transmit an accurate signal)^8^.

Contrary to this study’s hypothesis, anger and disgust did not systematically amplify all facial movements associated with pain. Instead, it was found that anger, disgust, and pain thus share almost indistinguishable facial representations. This finding is consistent with the results of some studies on pain communication. For instance, AUs typically associated with disgust and anger explained 64% of the variance in the prediction of pain AU frequency measured in patients undergoing blood sampling^27^. Also, stereotypical facial configurations of anger and disgust were observed on, respectively, 14% and 21% of patients undergoing a painful clinical assessment^26^. Anger and disgust are threat-related emotions that can be induced by stimuli in the environment with actual danger (e.g., Ref.^95^). Similarly to anger and disgust, some authors emphasize the threatening aspect of pain rapidly capturing attention when observing others^96^. A recent study^32^ found that a diversity of facial movements could trigger threat perception on others’ faces (particularly anger) or non-threat perception (e.g., sadness), as some facial movements reflect signal degeneracy (i.e., different facial movements eliciting the perception of the same affective state) or redundancy (i.e., similar facial movements eliciting the perception of the same affective state)^97^. This distinction could suggest that the facial configurations associated with anger, disgust, and pain have been evolutionarily optimized to share a similar representation so that individuals can effectively detect a threat even in a visually overstimulating environment and ensure human survival. In contrast to potential threat signals (e.g., fear), anger and disgust may provoke immediate pain-like behaviors to protect the body when facing an actual danger (i.e., fight-flight-or-freeze)^4,90,98,99^. Negative affective states can thus be described as dispositions to action: heightened vigilance and information gathering (e.g., in the context of fear) to the autonomic and motor responses to counter/escape threats (e.g., in contexts of anger, disgust, or pain)^100^. This interpretation supports the conception of some authors that emotions are functional states that contextually promote an adaptative set of actions^30^. Therefore, anger, disgust, and pain are threatening signals that share common facial representations when communicated to others.

Some facial movements were found to be more specifically associated with pain and were not modulated by the different affective contexts (i.e., AUs 6–7-43/45 and AU 12). The tightening of the orbital region muscles with eyes closing (i.e., AUs 6–7-43/45) was more intense for Pain scenarios than other scenarios on the VAs. This facial movement was identified as a distinctive feature of the stereotypical facial configuration of anger^24^, and as the AUs most frequently observed in different clusters of pain expressions^101^. It also has been linked with the sensory component (i.e., the intensity) of pain, which can be distinguished from the affective component (i.e., the unpleasantness) of pain^21^. Roy et al.^22^ found in their study that an optimized simulation model relies on the information of the eyes (inferior part of the *orbicularis*) to recognize pain from other emotions. The tightening of the orbital region muscles with eyes closing could thus inform about the sensory component of pain that is not shared with emotions. Furthermore, the lip corner pulling (i.e., AU 12), which is traditionally associated with smiling and positive affective states (e.g., Ref.^102^), was surprisingly found to be more intense on participants’ faces for Pain scenarios than other scenarios. Smiles may reflect discomfort in performing the task of expressing pain. They have also been observed repeatedly in experimental and clinical pain studies^103^. Kunz et al.^103^ suggest that rules of social display strongly modulate the expression of pain and that smiling during pain could aim to create a social bond with the observer to ensure support in the event of need. However, given the absence of the lip corner pulling on the VAs to represent pain, there is potentially an incongruent expectation of no smiles during painful events. This finding highlights the need to investigate the (potentially negative) impact of smiling during pain on observers in future studies.

Differences were found between the representation of facial configurations on the VAs and the participants’ faces. In addition to the specificity of certain facial movements for pain, the results show that, on the VAs, anger amplifies the furrowing of the brows (i.e., AU 4) and the nose wrinkling and upper lip raising (i.e., AUs 9–10), and disgust reduce the intensity of the facial movements associated with pain. The amplification effect of anger on facial movements associated with the affective component of pain is relevant to the prevalent theory about the effect of emotions on pain experience (i.e., motivational priming theory). This theory proposes amplification of pain experience by negative emotions with low-to-moderate levels of arousal but pain inhibition by high arousal negative emotions^3^. However, this theory cannot explain the reducing effect of disgust, which may instead reflect the context-dependent perception of affective states. For example, another affective state, such as amusement, could have been perceived from Disgust-Pain scenarios and have contributed to diminishing the effect of disgust on facial movements associated with pain. In a previous study^104^, participants watched amateur videos representing humorous lapses (to induce pure amusement), ambiguous bloopers (to induce mixed amusement and disgust), or accidents (to induce pure disgust). During these videos, electromyographic data was collected on the *corrugator supercilii* muscle regions (i.e., causing the furrowing of the brows, AU 4). The research team found that the *corrugator* activity was the most intense during disgusting films, followed by mixed films, and then amusing ones that were no different from the baseline. The amusing effect that could have emerged from Disgust-Pain scenarios representing ambiguous blooper situations (e.g., seeing someone with “brain freeze” symptoms while eating ice cream) might have influenced the results. Finally, chin raising (i.e., AU 17), rather than lip corner depressing (i.e., AU 15) found on the VAs, was found to be more intense on posed facial configurations for Sadness-Pain scenarios than in other scenarios. This finding is consistent with the results of Gosselin et al.^55^. In their study, participants frequently activated the chin raiser when the lip corner depressor was the target facial movement they had to voluntarily imitate.

In general, more disparities between facial configurations of affective states were found on the VAs than on the participants’ faces in the current study. The design of the interactive VAs application (*Expressive EEVEE*) had some facial movements integrated into the same slider to ease manipulation (e.g., upper lid raising, AU 5, and tightening of the orbital region muscles with eyes closing, AUs 6–7-43/45). This clustering might have accentuated the differences between affective states on the VAs (e.g., Fear-Pain vs. Pain). Despite this effect with the VAs, the sensorimotor representation measured through the participants’ faces was found to be not very intense and with small differences between affective states.

This distinction in visual and sensorimotor representations may reflect differences identified between posed and spontaneous facial configurations of pain. Posed facial configurations of pain (also called exaggerated expressions) appear to be dramatized or can represent an intensified version of spontaneous expressions (also called genuine expressions)^105^. However, observers tend to perceive these posed facial configurations as realistic and more painful than spontaneous ones^106^. As a result, posed facial configuration could reflect a more congruent manifestation of the shared visual representation than spontaneous configurations, thus promoting the accuracy of pain communication. The differences between visual and sensorimotor representations should be the topic of further study to determine the accuracy of pain communication between an expressor and a perceiver.

The differences in facial movements found on the VAs and the participants’ faces in the current study also support the idea that affective states are communicated through facial configurations by distinct (yet interrelated) mechanisms. In the study of Le Mau et al.^107^, images of actors portraying posed facial configurations elicited from short scenarios were classified using an unsupervised clustering approach based on the emotions perceived in these scenarios. The authors found that the classification of the actors’ facial poses differed from the typical facial configurations of emotion. Even when displayed deliberately by affective encoding experts (i.e., actors) under controlled conditions (i.e., reading scenarios in a photo studio), stereotypical facial behaviors could not be inferred. Furthermore, the recognition of a category of emotion from a facial configuration has been linked to the individual's visual representation of this category, which is subject to variations between individuals (e.g., Refs.^12,13,56,57^). Therefore, studying the variability of internal representations (e.g., visual, or sensorimotor) could shed light on the variability observed in encoding and decoding processes between people as well as between situations.

The SHAP analysis reveals that the classification of imitated and posed facial configurations could be achieved primarily based on the tightening of the orbital region muscles with eyes closing (i.e., AUs 6–7-43/45), the nose wrinkling and upper lip raising (i.e., AUs 9–10), and the lip corner pulling (i.e., AU 12). As predicted, these facial movements are easier to imitate voluntarily for the participants than other AUs^55^. Moreover, the intensity of facial movements was higher on imitated than posed facial configurations. These results suggest that conceptual information about affective states from reading scenarios elicits more intense facial movements than visual information about the facial configurations from observing expressive VAs. This possible interpretation is in accord with the findings of Le Mau et al.^107^, showing greater importance of emotional information from scenarios than the observation of facial movements alone to perceive the affective state of others. In a meta-analysis comparing the effect sizes of various affect induction procedures^108^, the reading of a story (δ = 1.80) has also a stronger effect size than the manipulation of the face (δ = 0.73). Therefore, conceptual information about the affective state is better than visual information about the facial configuration at inducing a certain affective state and facilitating its expression. This observation suggests that the presentation of facial configurations without further information on the context may not be sufficient to trigger some affect-related abilities, which could be relevant in certain clinical settings (e.g., social deficits in autism spectrum disorders).

Some limitations restrain the generalization of the results. The data was collected from a relatively small and homogenous sample of participants from a Canadian university that does not reflect all the culturally various ways to represent affective states through the face (e.g., Ref.^109^). This study offers an innovative empirical method that could be applied to questions regarding potential cultural and ethnic differences. Using an automated recognition algorithm of facial movements instead of manual coding could also be perceived as a limitation. The performance of automated algorithms in detecting AUs is not the same as the manual FACS coding (biserial correlation value of ± 0.80). Some AUs are better detected by these algorithms (e.g., AUs 2, 9, 17, and 25–26) than others (e.g., AUs 7, 20, and 23)^110^. The results from the direct comparison between representations on the VAs and the participants’ faces should be generalized with caution because the application of interactive VAs (*Expressive EEVEE*) was not developed around the same AUs intensity detection algorithm used on the participants’ faces (OpenFace) but rather on the expertise in FACS coding of a 3D animator^50^. The design of the sliders in the application and the fixed number of AUs included in this study should also be considered a limitation. Only facial movements frequently associated with pain and certain negative emotions were selected in the development of the application and from the detection algorithm. Consequently, all possible combinations of AUs could not be measured. Moreover, dynamic characteristics of facial movements could not be analyzed in this study, as little information is yet available in the literature on the dynamic characteristics of individual AUs to be integrated into virtual agents. Future studies should focus on these characteristics (e.g., by integrating the dynamic parameters of various facial movements into an interactive application of virtual agents).

In conclusion, this study highlights the complex relationships between pain and emotions when these states are communicated through the face. Contrary to what one might expect, the combined affective states of pain and emotions do not result in a simple addition of the two stereotypical facial configurations. Affective states related to a direct threat to the body (i.e., anger, disgust, and pain) share a similar facial representation, while those that present no immediate danger (i.e., fear and sadness) differ. Although visual and sensorimotor representations emerging from these states provide congruent affective information, they are differently influenced by factors associated with the communication cycle (e.g., the type of information to induce affective states and the role of context in encoding and decoding affective states). These findings stress the need to update current models of nonverbal communication of pain to include the other affective states and the different mechanisms (i.e., visual, sensorimotor, and conceptual) of the facial configurations that support social interactions.

The theoretical advances in pain communication could benefit some applied research domains, such as affective computing. For instance, machine learning models targeting the automatic detection of pain by the face should not only include the facial movements associated with pain but incorporate all the variety of nonverbal behaviors and contextual elements that convey affective content, including facial movements associated with other negative affective states. In doing so, the dynamic, multimodal, and multiplex nature of affective communication would be better integrated with the machine learning models. In the not-so-distant future, where some human-agent interactions will be more frequent and driven by artificial intelligence (e.g., for various applications in education and healthcare), detecting and simulating facial configurations perceived as pain will be an essential research focus that will benefit from including combined affective states. A clinically relevant research idea in the field of affective computing is the development of interactive virtual agents that could be used to dynamically and precisely measure the affective experience of non-communicative patients such as young children and older adults suffering from cognitive impairment (such as a “visually-interactive” Faces Pain Scale^111^).

Subsequent studies must address some unanswered questions regarding the distinct processes involved in communicating the combined affective states of pain and emotions to progress toward these future applications. For instance: Can two affective states be temporally overlapped on an expresser’s face (i.e., encoding process)? Which visual cues are associated with an observer's detection of a combined affective state (i.e., decoding process)? What are the similarities and differences between visual, sensorimotor, and conceptual representations of affective states through facial configurations? The accumulation of data on affective communication in multiple contexts, by mobile neurophysiological sensors, for example, will surely benefit our understanding of complex affective states and their validity when communicated nonverbally^7^. As Gilam et al. proposed^1^, the promising future of research on pain and emotions depends on bridging theoretical views and academic disciplines. With this in mind, this project aims to be part of the foundation of this innovative and exciting evolution in affective sciences.

### Supplementary Information

Supplementary Information.

## Data Availability

The datasets generated during and/or analyzed during the current study are available from the corresponding author upon reasonable request.
