# Supplementary material for: Facial representations of complex affective states combining pain and a negative emotion
Source: Sci Rep. 2024 May 22;14:11686. doi: 10.1038/s41598-024-62423-2 (PMC11111784; doi:10.1038/s41598-024-62423-2)
Supplement: Supplementary file 1 — Supplementary Information. [file 41598_2024_62423_MOESM1_ESM.pdf]

Supplementary Information for

Facial representations of complex affective states combining pain and a negative emotion

Marie-Hélène Tessier[1,2,3], Jean-Philippe Mazet[4], Elliot Gagner[1,2,3], Audrey Marcoux[1,2,3], & Philip L. Jackson[1,2,3*]

1. School of Psychology, Université Laval, Québec City, Canada
2. Centre for Interdisciplinary Research in Rehabilitation and Social Integration (Cirris), Québec City, Canada
3. Cervo Research Center, Québec City, Canada
4. Department of Computer Science and Software Engineering, Université Laval, Québec City, Canada

* **Corresponding author:** philip.jackson@psy.ulaval.ca

This PDF file includes:

Supplementary Appendices 1 to 8
Supplementary Figures S1 to S20
Supplementary Tables S1 to S8

Supplementary Information

Appendix 1. Scenarios

Table S1

Fifteen scenarios describing fictional characters in different daily life situations involving pain and a negative emotion (pain combined with either anger, disgust, fear, or sadness) or strictly involving pain.

Affective states	Original texts (French)	Unvalidated translation (English) [a]
**Anger-Pain**	Un individu pressé est furieux d'avoir oublié son porte-monnaie au magasin. D'un pas rapide, il descend les escaliers de son immeuble d'habitation. Tout à coup, il perd pied et déboule violemment les escaliers.	An individual in a hurry is furious to have forgotten their wallet at the store. They walk briskly down the stairs of their apartment building. Suddenly, they lose their footing and crash violently down the stairs.
**Anger-Pain**	Une personne entre dans une salle de jeu. Par mégarde, elle marche sur des jouets au sol qui lui créent une vive douleur. Elle fulmine contre les jouets qui ont été laissés traîner par les enfants.	A person enters a playroom. They accidentally step on toys lying on the floor, inflicting a sharp pain. They lash out at the toys left lying around by the kids.
**Anger-Pain**	Une personne a de l'animosité envers son collègue. Elle le provoque et se dispute avec lui. Son collègue la frappe au ventre et elle se plie en deux de douleur.	A person feels animosity towards their colleague. They provoke them and argue with them. Their colleague punch them in the stomach, causing them to double over in pain.
**Disgust-Pain**	Un individu se fait convaincre par son ami de manger un dessert glacé qui l'écœure. Aidé par les encouragements de son ami, il réussit à manger au complet son plat glacé. Cependant, en mangeant trop vite, un mal de tête lancinant apparaît.	An individual's friend convinces them to eat a frozen dessert that disgusts them. Aided by their friend's encouragement, they manage to eat the entire frozen dish. Eating too quickly, however, gives them a throbbing headache.
**Disgust-Pain**	Une personne fait une randonnée en montagne pendant quelques jours. Puisqu'elle est mal chaussée, ses pieds s'enflent et deviennent douloureux. En retirant ses chaussures, elle s'aperçoit qu'elle a de nombreuses ampoules répugnantes.	A person is hiking in the mountains for a few days. Their feet swell and become painful due to ill-fitting shoes. As they take off their shoes, they discover that they have numerous repugnant blisters.
**Disgust-Pain**	Un individu marche dans de l'herbe à poux. Puisque ses jambes lui démangent atrocement, il se gratte jusqu'au sang. Il est répugné par les	An individual is walking through ragweed. Severely itching, they scratch their legs until they bleed. They are repelled by the resulting blisters on their legs.

	cloques formées par la suite sur ces jambes.	
**Fear-Pain**	Une personne se tient sur le balcon de ses amis malgré sa peur des hauteurs. Elle entend soudainement des cris et s'appuie légèrement sur le garde-fou pour voir. Le garde-fou cède sous son poids et elle se fracture l'épaule en tombant d'un étage.	A person stands on their friends' balcony despite their fear of heights. They suddenly hear screams and lean lightly on the guardrail to see. The guardrail yields to the pressure of their weight causing them to fall and fracture their shoulder.
**Fear-Pain**	Un individu en camping est seul dans sa tente. En tentant de sortir de sa tente pour aller aux toilettes durant la nuit, il trébuche et se casse le nez. Il ne voit rien autour de lui et il est apeuré des bruits qu'il entend.	An individual is camping alone in their tent. Trying to get out of their tent to go to the toilet at night, they stumble and break their nose. They see nothing around them and are frightened by the noises they hear.
**Fear-Pain**	Un individu se fait du souci pour sa santé physique. Il va faire une marche pour se changer les idées. Il ressent alors une forte douleur à la poitrine et un engourdissement au bras gauche.	An individual is worried about their physical health. They go for a walk to clear their mind. They then feel an intense pain in their chest and numbness in their left arm.
**Sadness-Pain**	Un individu monte une montagne sous la pluie. Il glisse sur une roche mouillée et fracasse son genou au sol. À cause de sa blessure, il doit renoncer à sa randonnée, ce qui le désole.	An individual hikes a mountain in the rain. They slip on a wet rock and smash their knee on the ground. Due to their injury, they give up the hike, which saddens them.
**Sadness-Pain**	Seul chez lui, un individu se sent abandonné par sa famille. À cause d'une vieille blessure au genou, il a de la difficulté à se déplacer en dehors de chez lui. L'inflammation à son genou lui crée une vive douleur à chaque mouvement.	Alone at home, an individual feels abandoned by their family. An old knee injury makes leaving their house very difficult. The inflammation in their knee causes them sharp pain with every movement.
**Sadness-Pain**	Une personne recommence à jouer à son sport préféré, le tennis. Ses douleurs au dos qui persistent depuis quelque temps sont exacerbées en frappant la balle. Elle est attristée de réaliser qu'elle n'est plus en mesure de jouer au tennis.	A person resumes playing their favorite sport, tennis. Their lingering back pain is exacerbated every time they hit the ball. They are saddened to realize that they can no longer play tennis.
**Pain**	Une personne travaille à son bureau et cherche une feuille dans un tiroir. Elle referme rapidement le tiroir du bureau et s'écrase deux doigts. Elle se tortille de douleur sous la force de l'impact.	A person is working at their desk and is looking for a paper sheet in a drawer. They quickly close the desk drawer and crush two fingers. They writhe in pain from the force of the impact.

**Pain**	Un individu se gèle les pieds, car il est mal chaussé. Il s’empresse de retourner à la maison pour se réchauffer. En trempant ses pieds dans l’eau chaude, il ressent des crampes pendant le dégel.	An individual freeze their feet because they are poorly shod. They rush home to warm up. Soaking their feet in warm water, they experience cramps as they thaw.
**Pain**	Une personne est à l’urgence avec une plaie douloureuse sur le bras. Une infirmière s’occupe d’elle pour la soigner. L’onguent qu’elle applique lui fait ressentir une sensation de brûlure sur la peau.	A person in the emergency room has a painful arm wound. A nurse is tending to it. The ointment they apply causes a burning sensation on their skin.

[a] The scenarios have been validated in French and not in English. The translation from French to English is provided for example purposes only.

Appendix 2. Validation Study of the Scenarios

Objectives

This study's main objective was to create and validate short written scenarios describing different pain situations where a fictional character feels a negative emotion simultaneously. Five scenario categories have been created and validated: Anger-Pain (AP), Disgust-Pain (DP), Fear-Pain (FP), Sadness-Pain (SP), and Pain only (P). In order to validate the content of these scenarios, two research questions were asked. (R1) Compared to the other categories, was the targeted emotion or pain perceived as more intense for one category of scenarios? For example, was the perceived intensity of anger more intense in AP scenarios than in the DP, FP, SP, and P scenarios? (R2) For each scenario, were the targeted emotion and the pain perceived as more intense than other measures of emotions? For example, for one of the AP category scenarios, was the perceived intensity of anger and pain greater than the perceived intensity of sadness, fear, and disgust?

Hypotheses

Concerning the first research question (R1), it was predicted that:

(1) The perceived level of anger would be greater for the scenarios representing situations of pain combined with anger (AP, H 1.1).

(2) The level of perceived sadness would be greater for scenarios representing situations of pain combined with sadness (SP, H 1.2).

(3) The level of perceived fear would be greater for scenarios representing situations of pain combined with fear (FP, H 1.3).

(4) The level of perceived disgust would be greater for scenarios representing situations of pain combined with disgust (DP, H 1.4)

(5) The level of perceived pain would be equal for every category of scenarios (P, H 1.5).

For the second research question (R2), the predictions were that:

(1) At least three scenarios representing situations of pain combined with anger (AP) would have an equal level of anger and pain that would be superior to other measures of emotions (H 2.1).

(2) At least three scenarios representing situations of pain combined with sadness (SP) would have an equal level of sadness and pain that would be superior to other measures of emotions (H 2.2).

(3) At least three scenarios representing situations of pain combined with fear (FP) would have an equal level of fear and pain that would be superior to other measures of emotions (H 2.3).

(4) At least three scenarios representing situations of pain combined with disgust (DP) would have an equal level of disgust and pain that would be superior to other measures of emotions (H 2.4).

(5) All control scenarios of pain (P) would have a level of pain superior to the measures of emotions (H 2.5).

Method

Participants. The sample comprised 46 healthy participants between 18 and 48 years old (23.56 ± 6.79 years old, 10 males). Participants were recruited by emails sent to the institutional lists of students and employees of *Université Laval*. They were excluded if they reported having a neurological or psychiatric disorder or a pain condition, worked with people suffering from a pain condition (e.g., health workers who are exposed frequently to pain expressions), or had previously participated in a study on pain expressions from our research laboratory. This

validation study was approved by the *Centre intégré universitaire de santé et de services sociaux (CIUSSS) de la Capitale-Nationale*'s Ethics Committee (#2020-1824). Informed consent was obtained by electronic signature from each participant, and their participation granted them eligibility to enter a draw with the chance to win a 50 $CAD gift card.

Creation of scenarios. The creation and selection of the scenarios for the validation study were completed in many steps described in Table S2. These steps aimed to obtain the best scenarios to induce the study's participants to specific affective states. The scenarios were divided into five categories according to the intended induced affective state: pain (control condition: P) and a combination of pain with anger, sadness, fear, and disgust (experimental conditions: AP, SP, FP, and DP).

Table S2

Description of the procedure to validate the scenarios.

	Category of scenarios				
	**Pain (P)**	**Anger + Pain (AP)**	**Sadness + Pain (SP)**	**Fear + Pain (FP)**	**Disgust + Pain (DP)**
**Step 1**: Creation of 200 scenarios	40 scenarios	40 scenarios	40 scenarios	40 scenarios	40 scenarios
**Step 2**: In-house validation with a member of the laboratory					
**Step 3**: Selection of 100 scenarios (200 → 100) [a]	20 scenarios	20 scenarios	20 scenarios	20 scenarios	20 scenarios
**Step 4**: External validation with a member of the community					
**Step 5**: Selection of 75 scenarios (100 → 75) [a]	15 scenarios	15 scenarios	15 scenarios	15 scenarios	15 scenarios
**Step 6**: In-house validation with some members of the laboratory					
**Step 7**: Selection of 50 scenarios (75 → 50) [a]	10 scenarios	10 scenarios	10 scenarios	10 scenarios	10 scenarios
**Step 8**: Final validation with members of the university community through an online study					
**Step 9**: Selection of 15 scenarios (50 → 15) [a]	3 scenarios	3 scenarios	3 scenarios	3 scenarios	3 scenarios

[a] For these steps, new scenarios could be created if the number of selected scenarios did not reach the target number or significant changes could be made to pre-existing scenarios based on participants' comments.

The first step consisted of creating 200 scenarios. Using a keyword search on pain and emotions, the principal investigator (MHT) and two research assistants drafted these scenarios according to the following criteria. Each scenario involved a character living different situations of daily life, combining physical pain and a negative emotion. Two gender-neutral terms were used to designate the character: individual and person. The scenarios were constructed from three

simple sentences containing 5 to 15 words for a total of 15 to 45 words per scenario. Each sentence offered information about the scenario's context (Context), the action leading to the pain and the painful feeling (PainExp), or the emotion following or preceding the pain (Emotion). The sentence about the Context described the place, the time, the objects, or the presence or absence of other characters in the scenarios. The sentence about Pain varied depending on the type of pain (acute or chronic, electrical, hot/cold, pressure, ischemic, etc.), the intensity of pain (weak to strong), and the area of pain (head, neck, shoulders, jaw, eyes, arms, stomach, belly, legs, feet, etc.). The sentence about the Emotion described one of four affective states simultaneously felt with pain by the character: anger, sadness, fear, and disgust. For the control scenarios, this sentence gave additional information about the pain the character feels. The sentences were organized according to two structures: the sentence about the Emotion preceded the one about Pain (Emotion → Context → PainExp), or the sentence about the Emotion followed the one about Pain (Context → PainExp → Emotion). These two types of structures were counterbalanced among the created scenarios. Here are three examples of AP, DP, and P scenarios, respectively, that were created during the first step:

(a) "A person is in the emergency room for several hours. They are waiting due to a minor burn on their hand. They are exasperated at the sight of other patients seeing the doctor before them."

(b) "A person gags at the sight of the remnants of a rotten egg in their locker. Smelling the odour emanating from the locker, they quickly close the door. As they close the door, they get their fingers stuck in the crack."

(c) "A person is driving at night. They are suffering from an excruciating migraine that day. The lights from the other cars accentuate the pain."

Following the creation of 200 scenarios, the second step was to create an online questionnaire using the *Google Form* platform to collect a laboratory member's evaluation of the scenarios' affective content (see next section for the evaluation description). The third step was to select the best 20 scenarios per category. The scenarios with the greatest levels of pain and targeted emotion and the other emotions' weakest levels were chosen. Concerning the control scenarios (P), those with the greatest level of pain and the weakest level of emotions were selected.

After selecting the best 100 scenarios, the fourth step was to create a new online questionnaire using the *Google Form* platform to collect a community member's evaluation of the scenarios' affective content. The fifth step was to select the best 15 scenarios per category according to the same procedure used in the third step. After selecting the best 75 scenarios, the sixth step was to create a new online questionnaire using the *Google Form* platform to collect four laboratory members' evaluations of the affective content. The seventh step was to select the ten best scenarios according to the same procedure used in the third step.

The 50 resulting scenarios were finally evaluated online in the validation study. The two last steps (eighth and ninth) consist of the validation study's design, implementation, and data analysis. The next sections are a detailed explanation of the evaluation method and the procedure for this validation.

Evaluation of the scenarios' affective content. In order to evaluate the scenarios' affective content, the participants were asked three questions at every step of validation for each scenario. The participants had to evaluate the intensity of anger, sadness, fear, disgust, and pain expressed by the character in the scenario. In the questionnaires for steps 2, 4, and 6, the five affective states were rated using a Likert scale going from 1 (minimum intensity) to 5 (maximum

intensity) and an additional box of 0 if the perceived affective state was absent. The participants were required to click on one of the six boxes to indicate their answers on each Likert scale. For the validation study, five affective states were rated using five different visual analog scales (VAS) presented simultaneously, ranging from no affective state (0) to the maximum of an expressed affective state (100). The order of presentation of the VAS was random for each participant. The participants needed to move the marker on the line to indicate their answers on each VAS. The initial position of the marker on the line was random for each try. The participants could also indicate any other emotion(s) they thought the character could express. A box was left at the participants' disposition to put down their idea(s). The participants could share comments or constructive ideas for each scenario by writing them in the appropriate space. Figure S1 shows how the questions were presented in the validation study.

Procedure

This online validation study lasted approximately 30 minutes. A post-doctoral fellow in our laboratory helped create the web platform (with Flask, a Python web framework), allowing the study to occur online. In order to participate in the study, the participants had to click on a hyperlink provided in the recruitment email that would lead them directly to the home page using their internet navigator. A preamble on the home page provided general information concerning the study. The following pages asked participants to provide certain personal information to verify the inclusion and exclusion criteria. Then, the informed consent questionnaires were presented. Next, the participants had to answer the laboratory's questionnaire concerning their socio-demographic information and experience with virtual characters.

The task began with an instructions page and four practice trials. Each trial was presented on a page divided into four sections (see Figure S1). The first section presented the scenario, and

the three others each presented a question that needed to be answered by the participants. It was specified to the participants that they needed to answer each VAS presented in the first question but that the two other questions were optional. The 50 trials containing the 50 scenarios were presented randomly to the participants.

In a hurry, an individual is furious to have forgotten their wallet at the store. At a fast pace, they walk down the stairs of their residential building. Suddenly, they lose footing and tumble violently down the stairs.

1. In your opinion, to what intensity are the affective states below expressed by the character in this scenario?
   Anger VAS: *No anger expressed* < ----◉------------------- > *Maximum anger expressed*
   Sadness VAS: *No sadness expressed* < -------------------◉---- > *Maximum sadness expressed*
   Fear VAS: *No fear expressed* < -----------◉------------ > *Maximum fear expressed*
   Disgust VAS: *No disgust expressed* < -----------------------◉ > *Maximum disgust expressed*
   Pain VAS: *No pain expressed* < -----------------------◉ > *Maximum pain expressed*
2. In your opinion, does the character in this scenario express other emotion(s) than the options offered in the previous question? If so, write your idea(s) here.
3. If you have any comments or ideas for improving this scenario, write them here.

Figure S1. Example of a scenario's presentation and the questions asked during the validation study.

The last part of the validation study allowed the participants to share their general comments about the study. They could answer three questions:

(1) “Did you find the scenario evaluation difficult? If so, what did you find difficult to do?”

(2) “According to you, did the described scenarios represent situations that could happen in reality? If not, what did you find unrealistic about the scenarios?”

(3) “Do you have other comments you wish to share with us?”

The last page of the online study indicated that the participants were now eligible for the draw as they had completed the study.

Results

The first research question (R1) was to compare the scenario categories according to the intensity of each emotion and pain VAS. In order to do this, five repeated measures ANOVA tests were carried out on the five VAS (anger, sadness, fear, disgust, and pain). The independent variable corresponded to the five scenario categories (AP, SP, FP, DP, and P). Firstly, the ANOVA on the anger VAS showed significant differences between the scenario categories, $F(2.43, 106.82) = 206.74$, $p < .001$, $\eta^2_p = .83$. More anger was perceived in the AP scenarios than in other scenarios (SP, FP, and P, *p*s < .001). Secondly, the ANOVA on the sadness VAS showed significant differences between the scenario categories, $F(1.58, 69.71) = 432.72$, $p < .001$, $\eta^2_p = .91$. More sadness was perceived in the SP scenarios than in other scenarios (AP, FP, DP, and P, *p*s < .001). Thirdly, the ANOVA on the fear VAS showed significant differences between the scenario categories, $F(1.93, 84.98) = 438.82$, $p < .001$, $\eta^2_p = .91$. More fear was perceived in the FP scenarios than in the other scenarios (AP, SP, DP, and P, *p*s < .001). Fourthly, the ANOVA on the disgust VAS showed significant differences between the scenario categories, $F(1.42, 62.55) = 255.47$, $p < .001$, $\eta^2_p = .85$. More disgust was perceived in the DP scenarios than in other scenarios (AP, SP, FP, and P, *p*s < .001). Figure S2 presents the results from the four ANOVAs on the anger (red), sadness (blue), fear (violet), and disgust (green) VAS.

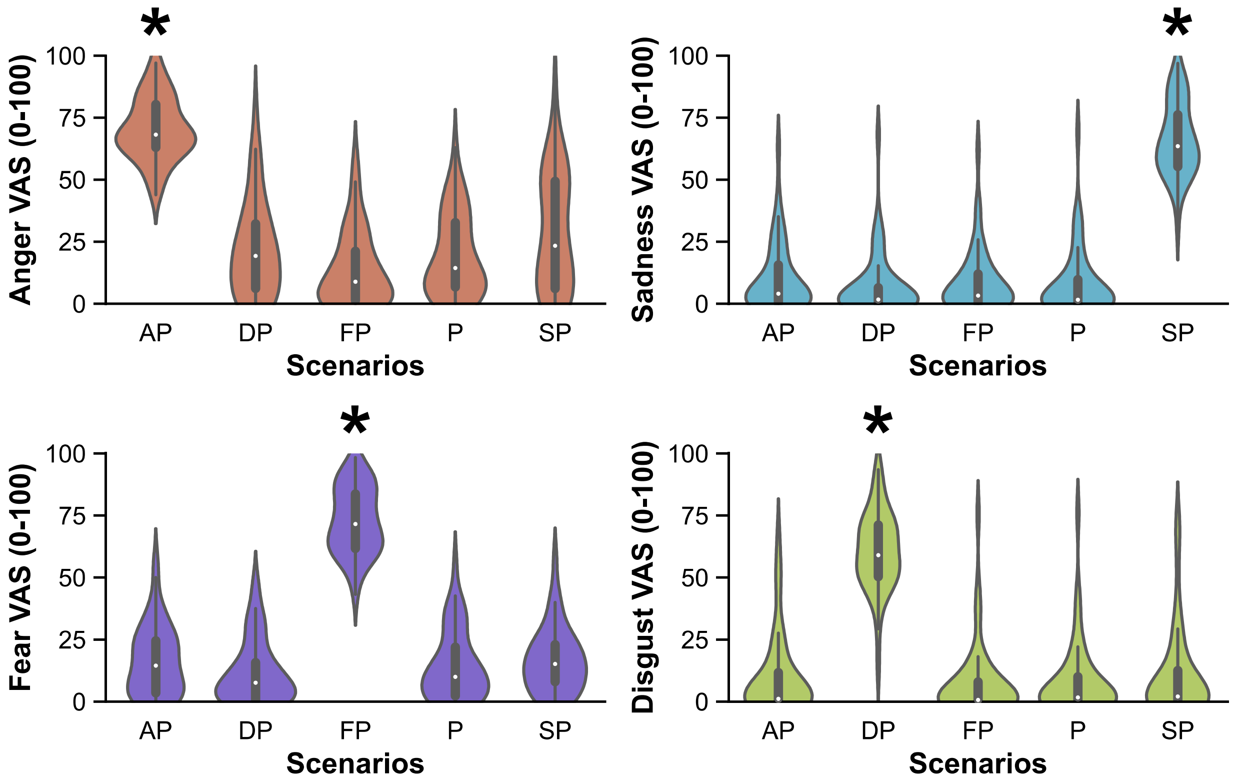

Figure S2. Data distribution of anger (red), sadness (blue), fear (purple), and disgust (green) visual analogue scales (VAS; transposed on numerical scales ranging from 0 to 100) according to the type of scenarios. The whiskers present the minimum and maximum values, the vertical length of the box presents the interquartile range, and the white circle within the box presents the median. AP: anger-pain; DP: disgust-pain; FP: fear-pain; P: pain; SP: sadness-pain. * *p*s < .001

Finally, the ANOVA on the pain VAS showed significant differences between the scenario categories, $F(3.00, 131.80) = 19.03$, $p < .001$, $\eta^2_p = .30$. The control scenarios (P) were indistinguishable from AP ($p = .017$) and FP ($p = .654$) regarding the perceived pain level intensity. However, more pain was perceived in the P scenarios compared to the DP and SP scenarios, *p*s < .001. Figure S3 presents the results from the ANOVA on the pain VAS.

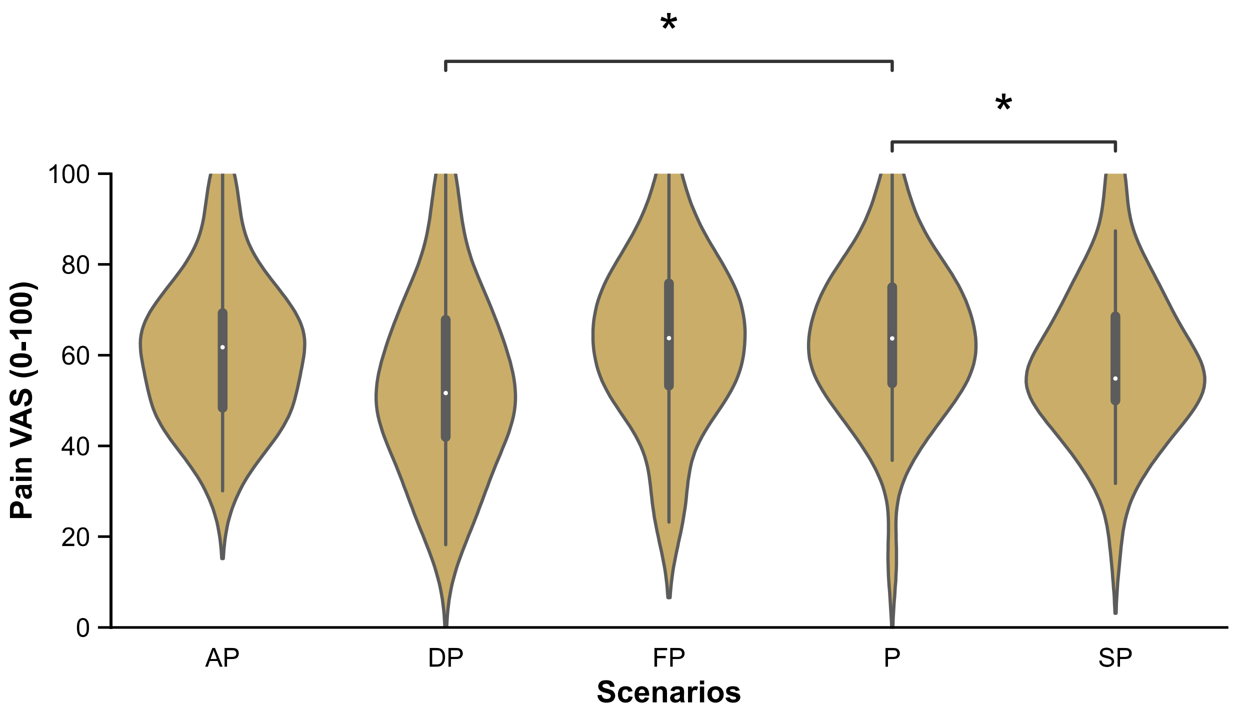

Figure S3. Data distribution of pain visual analogue scale (VAS; transposed on a numerical scale ranging from 0 to 100) according to the type of scenarios. The whiskers present the minimum and maximum values, the vertical length of the box presents the interquartile range, and the white circle within the box presents the median. AP: anger-pain; DP: disgust-pain; FP: fear-pain; P: pain; SP: sadness-pain. * $p < .001$

The second research question (R2) was to compare the intensity of the emotions and pain VAS for each scenario (e.g., anger vs. pain intensities in all AP scenarios). In order to do so, a repeated measures ANOVA test was carried out on each scenario. The independent variable corresponded to the five VAS categories (anger, sadness, fear, disgust, and pain). Firstly, all ANOVAs on the AP scenarios showed significant differences between the emotions and pain VAS, *p*s < .001. Compared to the other VAS, more anger and pain were perceived in the AP01, AP02, and AP09 scenarios. Furthermore, compared to the other VAS, more anger was perceived in the AP03, AP04, AP05, AP06, and AP10 scenarios, and more pain was perceived in the AP07 scenario (see Figure S4).

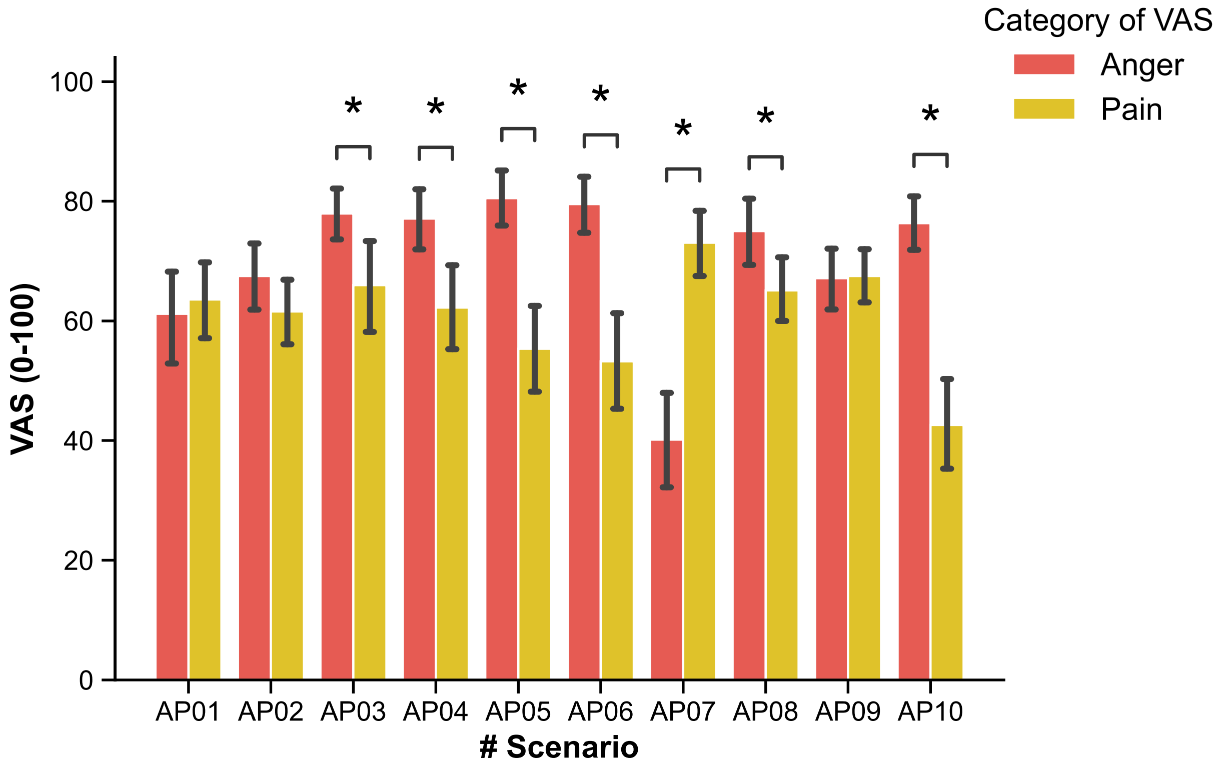

Figure S4. Means of the ten Anger-Pain (AP) scenarios on anger and pain visual analogue scales (VAS; transposed on numerical scales ranging from 0 to 100). The error bars indicate a 95% CI. * $p < .0025$

Secondly, all ANOVAs on the SP scenarios showed significant differences between the emotions and pain VAS, *p*s < .001. Compared to the other VAS, more sadness and pain were perceived in the SP01, SP05, SP06, SP08, SP09, and SP10 scenarios. Furthermore, compared to the other VAS, more sadness was perceived in the SP02, SP03, SP04, and SP07 scenarios (see Figure S5).

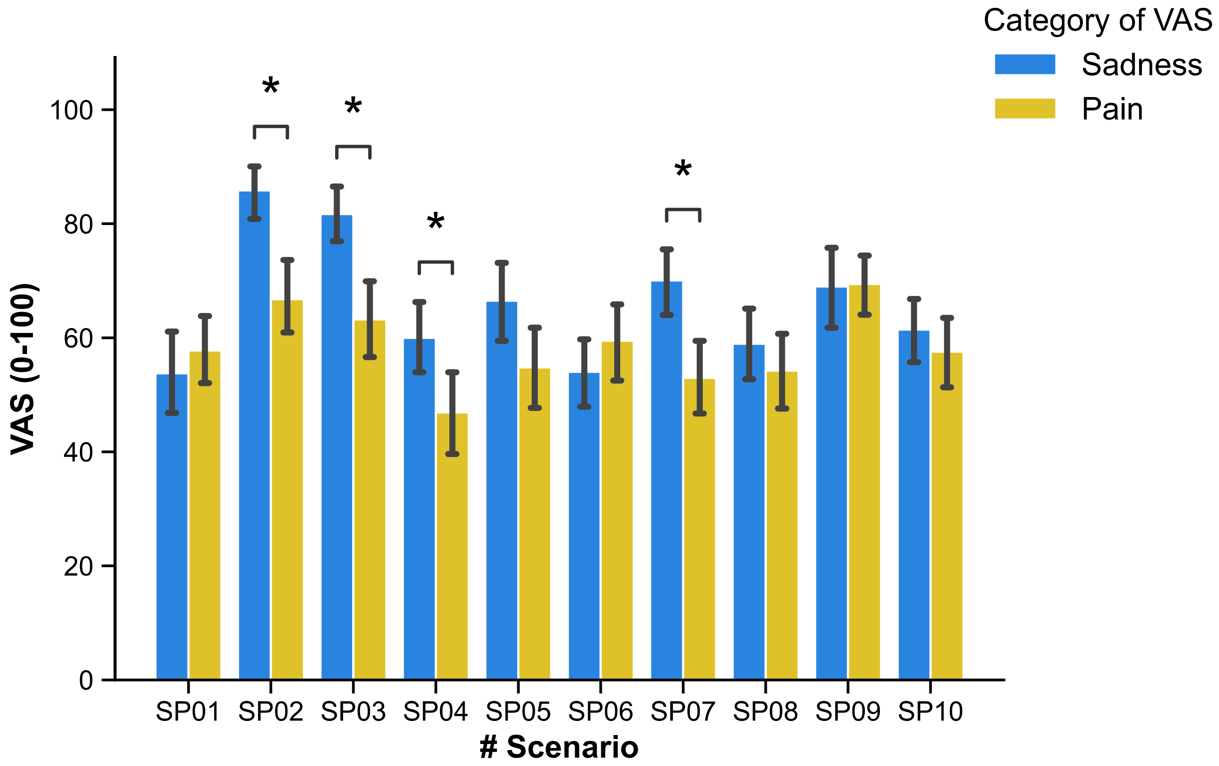

Figure S5. Means of the ten Sadness-Pain (SP) scenarios on sadness and pain visual analogue scales (VAS; transposed on numerical scales ranging from 0 to 100). The error bars indicate a 95% CI. * $p < .0025$

Thirdly, all ANOVAs on the FP scenarios showed significant differences between the emotions and pain VAS, *p*s < .001. Compared to the other VAS, more fear and pain were perceived in the FP01, FP02, FP03, FP04, FP09, and FP10 scenarios. Moreover, compared to the other VAS, more fear was perceived in the FP05, FP06, FP07, and FP08 scenarios (see Figure S6).

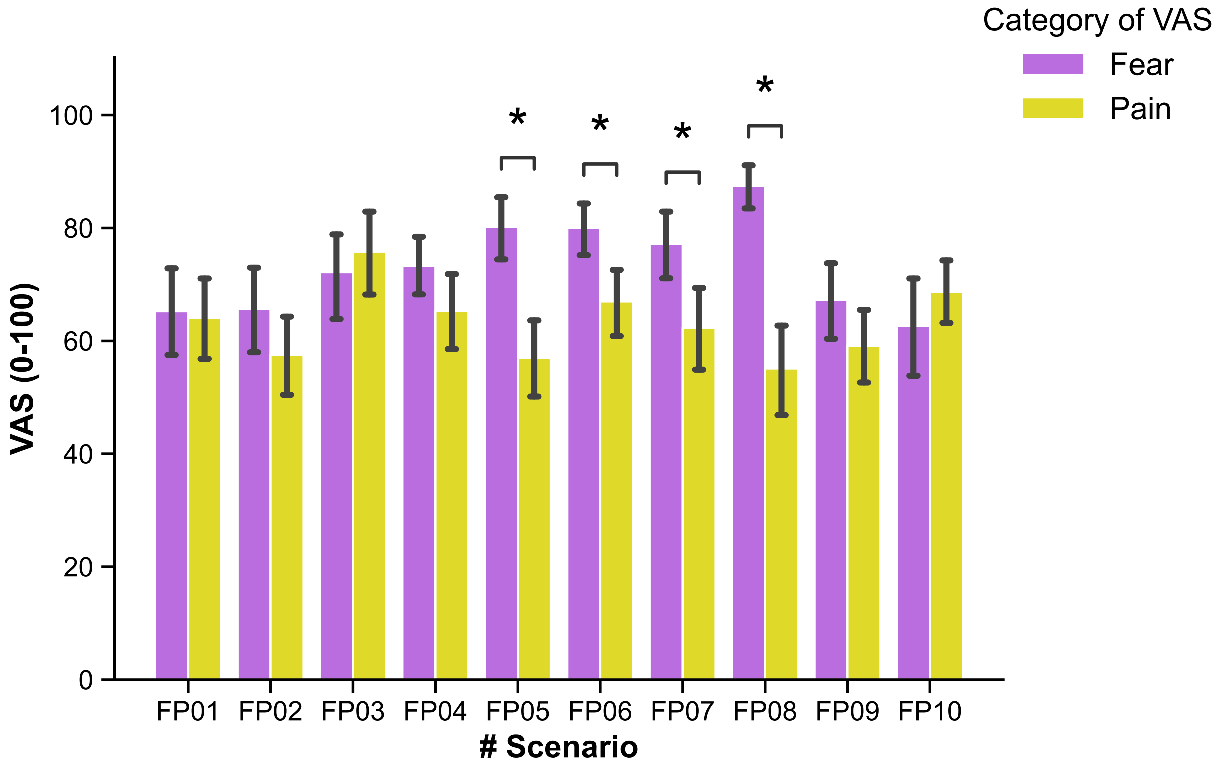

Figure S6. Means of the ten Fear-Pain (FP) scenarios on fear and pain visual analogue scales (VAS; transposed on numerical scales ranging from 0 to 100). The error bars indicate a 95% CI. * $p < .0025$

Fourthly, all ANOVAs on the DP scenarios show significant differences between the emotions and pain VAS, *p*s < .001. Compared to the other VAS, more disgust and pain were perceived in the DP01, DP05, DP06, and DP09 scenarios. Furthermore, compared to the other VAS, more disgust was perceived in the DP02, DP04, and DP08 scenarios, and more pain was perceived in the DP07 scenario (see Figure S7). Besides, compared to the other VAS, more pain and anger were perceived in the DP03 scenarios, whereas more disgust, pain, and anger were perceived in the DP10 scenario (results not illustrated in Figure S7).

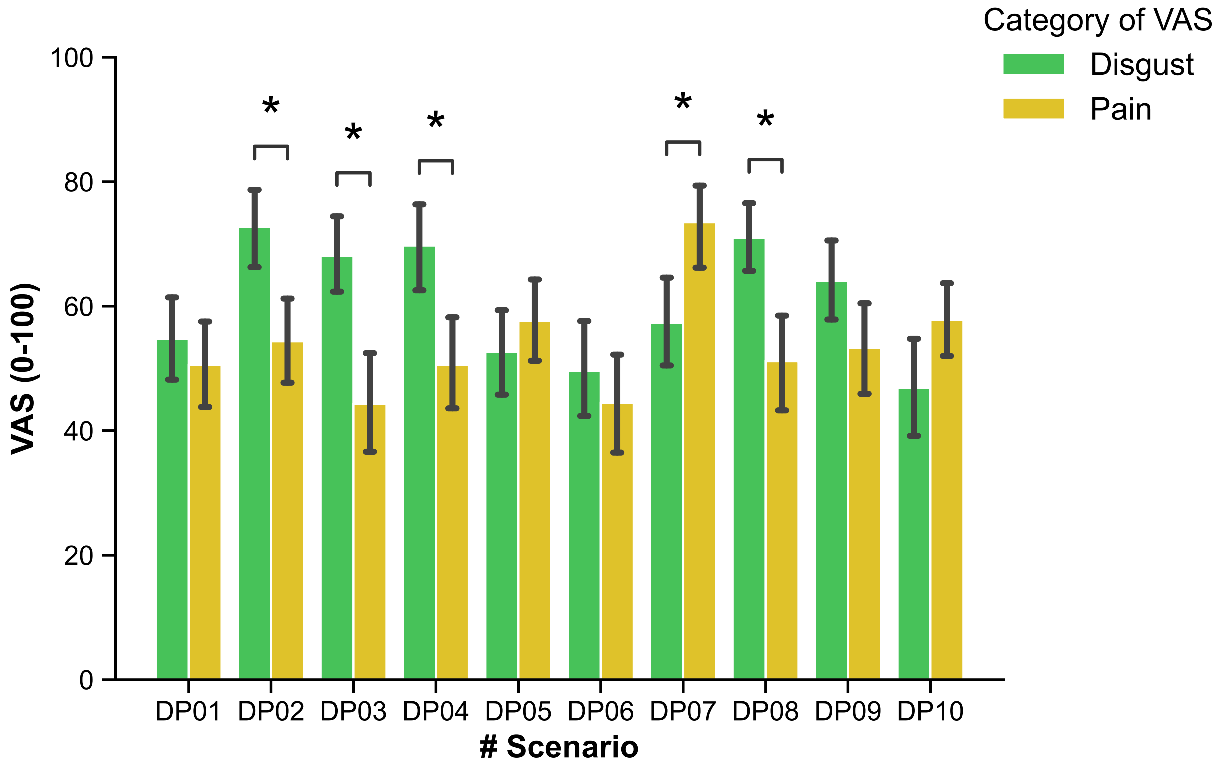

Figure S7. Means of the ten Disgust-Pain (DP) scenarios on disgust and pain visual analogue scales (VAS; transposed on numerical scales ranging from 0 to 100). The error bars indicate a 95% CI. * $p < .0025$

Lastly, all ANOVAs on the P scenarios showed significant differences between the emotions and pain VAS, *p*s < .001. Compared to the other VAS, more pain was perceived in all the P scenarios being P01, P02, P03, P04, P05, P06, P07, P08, P09, and P10 (see Figure S8).

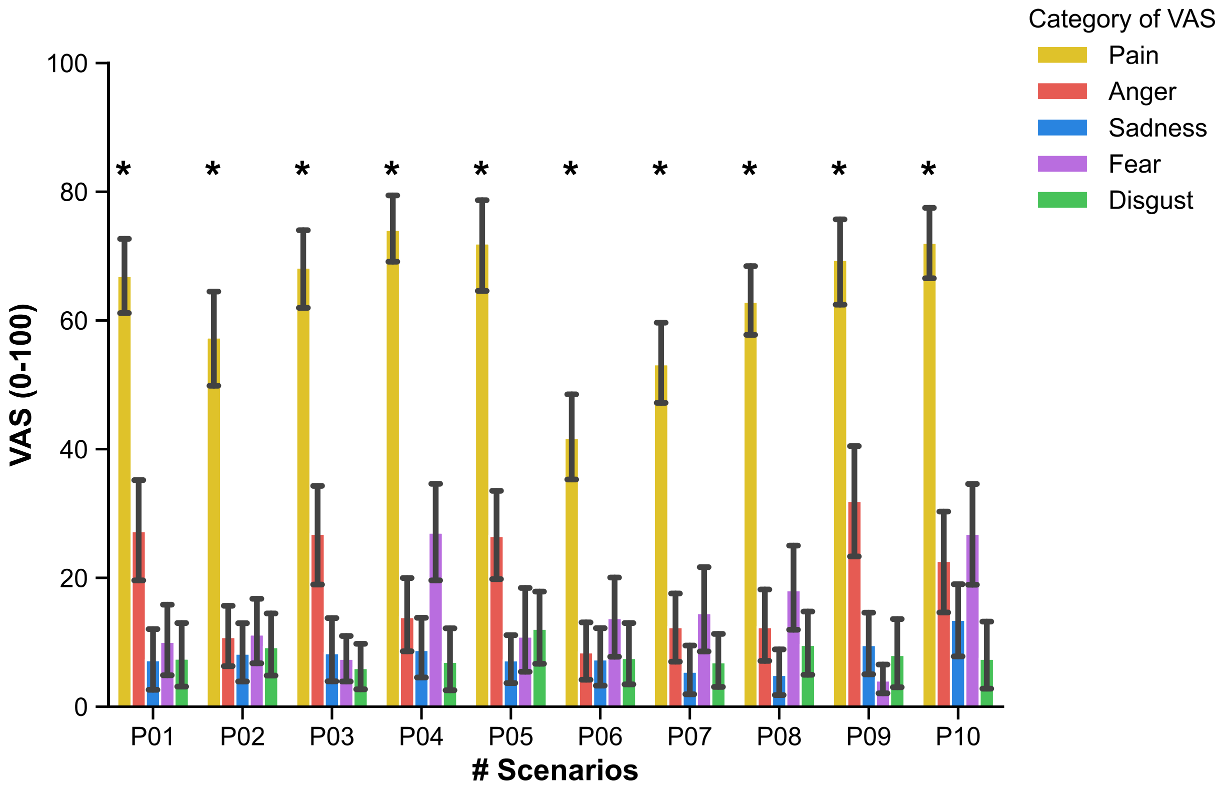

Figure S8. Means of the ten control scenarios (Pain only; P) on pain, anger, sadness, fear, and disgust visual analogue scales (VAS; transposed on numerical scales ranging from 0 to 100). The error bars indicate a 95% CI. * $ps < .0025$

Discussion

This study aimed to create and validate short written scenarios describing different situations of pain in which a fictional character feels a negative emotion simultaneously. The first research question (R1) was to determine if the perceived intensity of the targeted emotion or the pain was greater for one category of scenarios compared to the other scenario categories. The first four hypotheses were confirmed. The perceived level of anger is greater for the scenarios representing situations of pain combined with anger (AP, H 1.1). The level of perceived sadness is greater for scenarios representing situations of pain combined with sadness (SP, H 1.2). The level of perceived fear is greater for scenarios representing situations of pain combined with fear

(FP, H 1.3). The level of perceived disgust is greater for scenarios representing situations of pain combined with disgust (DP, H 1.4). Thus, the AP, SP, FP, and DP scenarios accurately describe the targeted emotions of anger, sadness, fear, and disgust, respectively.

It was also predicted that the level of perceived pain would be equal among the scenario categories (P, H 1.5). However, the results showed that, compared to the scenarios representing pain combined with sadness and disgust (SP and DP), more pain was perceived from the control scenarios (P) and the scenarios representing pain combined with anger and fear (AP and FP). This result might be explained by the inherent arousal level of the emotions and their interaction with pain. Previous studies have described the effect of valence and arousal of emotions on pain (for a summary, see [3] in the main text). Thus, low-to-medium-arousal negative emotions seem to ease the pain felt. Emotions with an inherently higher arousal (anger and fear) can increase the perceived pain compared to emotions with lower arousal (sadness and disgust). Given that the differences between scenario types on pain VAS were relatively small (M_{AP} = 60.99, M_{DP} = 53.59, M_{FP} = 63.10, M_{P} = 63.88, and M_{SP} = 58.26), the effect of these differences could only have a minimal effect on the creation of the main study's facial configurations.

The second research question (R2) was to determine whether the pain and targeted emotion in each scenario from the scenario categories were perceived as more intense than other measures of emotions. The five hypotheses were confirmed. At least three scenarios representing situations of pain combined with anger (AP01, AP02, and AP09) had an equal level of anger and pain that was superior to the other measures of emotions (H 2.1). Six scenarios representing situations of pain combined with sadness (SP01, SP05, SP06, SP08, SP09, and SP10) had an equal level of sadness and pain that was superior to the other measures of emotions (H 2.2). Six scenarios representing pain combined with fear (FP01, FP02, FP03, FP04, FP09, and FP10) had

an equal level of fear and pain that was superior to the other measures of emotions (H 2.3). Four scenarios representing situations of pain combined with disgust (DP01, DP05, DP06, and DP09) had an equal level of disgust and pain that was superior to the other measures of emotions (H 2.4). Finally, all the control scenarios of pain (P01 to P10) had a level of pain superior to the measures of emotions (H 2.5). Table S3 summarizes the final category of all the scenarios.

Table S3

Final category of the 50 scenarios following the validation study.

# Scenarios	Final category
AP01*	**Anger-Pain**
AP02*	**Anger-Pain**
AP03	Anger
AP04	Anger
AP05	Anger
AP06	Anger
AP07	Pain
AP08	Anger
AP09*	**Anger-Pain**
AP10	Anger
SP01	**Sadness-Pain**
SP02	Sadness
SP03	Sadness
SP04	Sadness
SP05	**Sadness-Pain**
SP06*	**Sadness-Pain**
SP07	Sadness
SP08	**Sadness-Pain**
SP09*	**Sadness-Pain**
SP10*	**Sadness-Pain**
FP01	**Fear-Pain**
FP02	**Fear-Pain**
FP03*	**Fear-Pain**
FP04*	**Fear-Pain**
FP05	Fear
FP06	Fear
FP07	Fear
FP08	Fear
FP09	**Fear-Pain**
FP10*	**Fear-Pain**
DP01*	**Disgust-Pain**
DP02	Disgust
DP03	Disgust > Pain = Anger [a]
DP04	Disgust
DP05*	**Disgust-Pain**
DP06	**Disgust-Pain**
DP07	Pain
DP08	Disgust
DP09*	**Disgust-Pain**
DP10	Pain = Disgust = Anger [a]
P01	**Pain**
P02	**Pain**
P03*	**Pain**
P04	**Pain**
P05	**Pain**
P06	**Pain**
P07*	**Pain**
P08*	**Pain**
P09	**Pain**
P10	**Pain**

[a] See the Results section for more information on the results obtained for these scenarios.
[*] The fifteen scenarios were chosen for the main study.

Appendix 3. Validation of the Virtual Agents

This is a summary of the results of the validation study. For more details, see:

Marcoux, A., Tessier, M.-H., Bélanger, C. & Jackson, P. L. Validation Experiments of Digital Characters from the EEVEE Platform: Experiment 1. (2023) doi:10.17605/OSF.IO/WQ9CS.

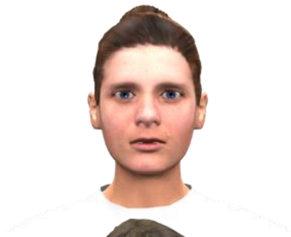

Female virtual agent
- Perceived age [a] = 25.16 ± 6.92 years old
- Perceived gender [b] = 17.14 ± 27.77 out of 100
- Perceived realism [c] = 52.29 ± 21.03 out of 100

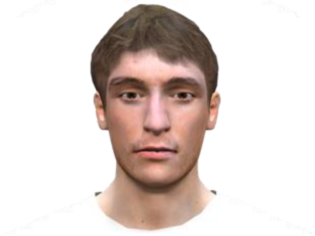

Male virtual agent
- Perceived age [a] = 23.87 ± 5.18 years old
- Perceived gender [b] = 97.36 ± 6.74 out of 100
- Perceived realism [c] = 71.31 ± 19.77 out of 100

Information about the sample
- Total of 363 participants
- Age = 35.33 ± 18.32 years old (between 18 and 93 years old)
- 28.7% reported being male, 71.0% female, and one participant preferred not to say
- 59.60% had a CÉGEP [d] or undergraduate degree
- 93.9% identified themselves as White, Caucasian, Anglo, European
- 60.8% were students, 25.7% were workers, and 13.5% were retirees
- 78.7% played video games and spent an average of 5.05 ± 6.36 hours per week playing video games

[a] Perceived age: Visual analog scale from 0 to 100 years.
[b] Perceived gender: Visual analog scale from Feminine [0] to Masculine [100], < 50 = Female, and > 50 = Male.
[c] Perceived realism: Visual analog scale from Not at all realistic [0] to Very realistic [100]. Based on the Uncanny Valley theory [1], the realism of virtual agents' appearance should not be inconsistent with the realism of their movements to avoid an eeriness reaction [2]. Therefore, the appearance of the virtual agents was intended to be moderately realistic in order to match the linear time course of their facial movements (which probably do not perfectly represent reality).
[d] i.e., college: first two or three years of post-secondary education in the province of Québec (Canada).

Appendix 4. Virtual Agents' Application (*Expressive EEVEE*)

a)

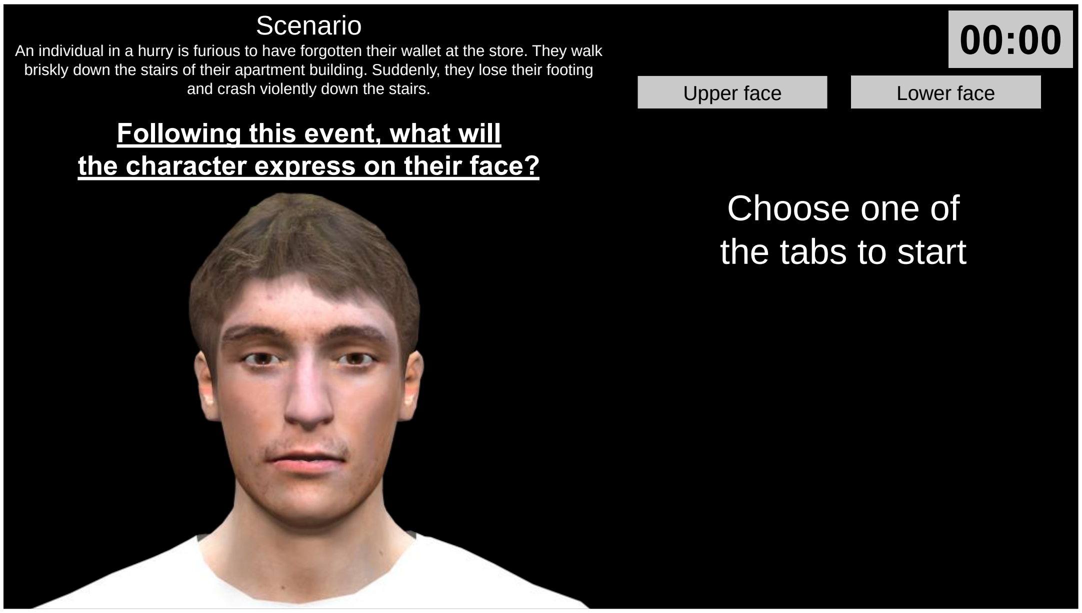

b)

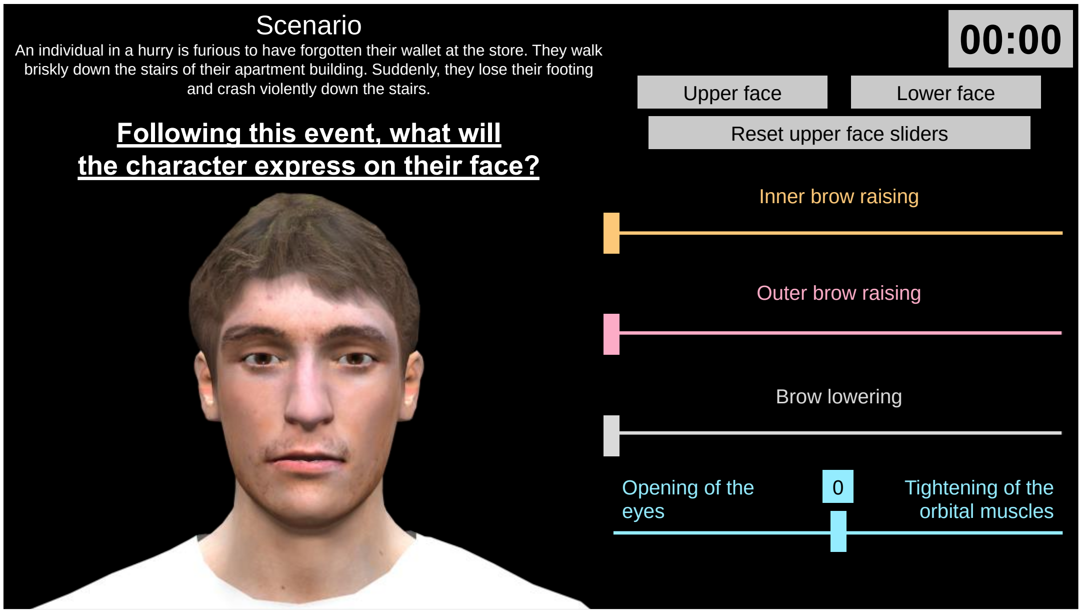

c)

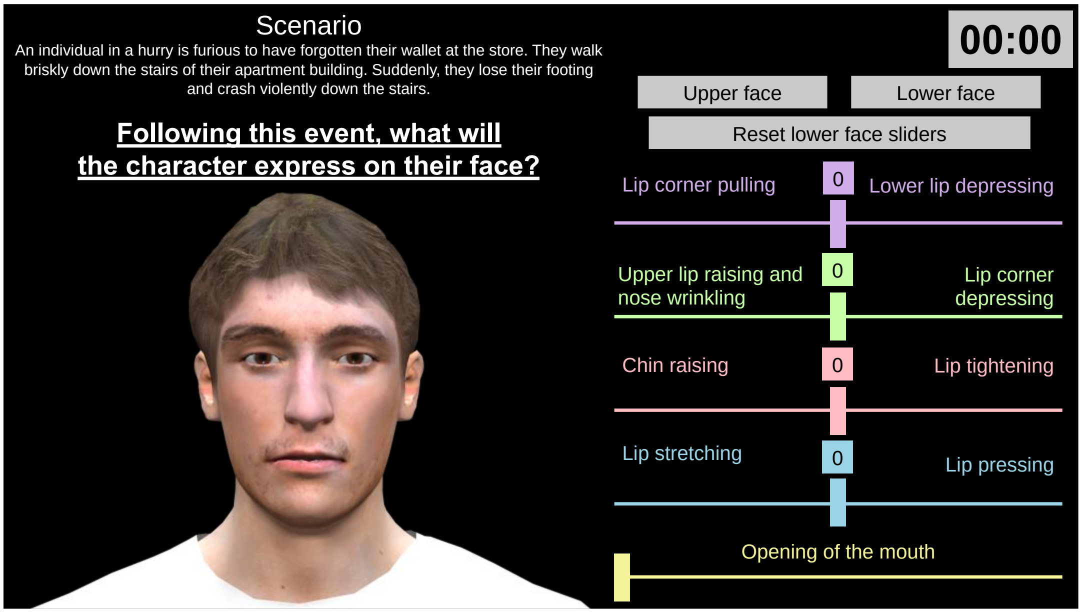

Figure S9. Representation of the three screens of the virtual agents' application (Expressive EEVEE). The text of the interface was translated from French to English in the Figure. *a)* Front screen at the beginning of each trial, where participants had to choose one of the tabs; *b)* Screen showing the upper face sliders after clicking on the "Upper face" tab; *c)* Screen showing the lower face sliders after clicking on the "Lower face" tab. The scenario, the question, and the virtual agent (male or female) were always located on the left of the screen. On the right, the timer showed the passing time (min:sec) and two grey buttons could be clicked to show the upper or lower face sliders, and a third grey button could be used to reset all sliders of the tab. A reset button ("0") was also available for each bidirectional slider.

Table S4

Associations between AUs and sliders on virtual agents (Expressive EEVEE).

Slider	Left end	Middle of the scale	Right end
		**Upper face**	
#1 Inner brow raising	Neutral face	N.A.	AU 1 (inner brow raising)
#2 Outer brow raising	Neutral face	N.A.	AU 2 (outer brow raising)
#3 Brow lowering	Neutral face	N.A.	AU 4 (brow lowering)
#4 Opening of the eyes and tightening of the orbital muscles	AU 5 (upper lid raising)	Neutral face	AUs 6 (cheek raising) – 7 (lid tightening) – 43 (eye closed)
		**Lower face**	
#5 Lip corner pulling and lower lip depressing	AU 12 (lip corner pulling)	Neutral face	AU 16 (lower lip depressing)*
#6 Upper lip raising with nose wrinkling and lip corner depressing	AUs 9 (nose wrinkling) – 10 (upper lip raising)	Neutral face	AU 15 (lip corner depressing)
#7 Chin raising and lip tightening	AU 17 (chin raising)	Neutral face	AU 23 (lip tightening)
#8 Lip stretching and lip pressing	AU 20 (lip stretching)	Neutral face	AU 24 (lip pressing)*
#9 Opening of the mouth	Neutral face	N.A.	AUs 25 (lips part) – 26 (jaw drop)

* Not included in the analyses (see *Expressive EEVEE* section in the manuscript for more details).

Appendix 5. Machine Learning Analyses

This section provides technical details and hyperparameters to reproduce the machine learning models (i.e., Multilayer Perceptron, MLP). The confusion matrices of the models, which provide a more detailed measure of the performance of their performance, are also presented. Additionally, visualizations of the classification models' input data (i.e., Principal Component Analysis, PCA) are included, offering insights into the task's classification difficulty.

Training strategy common to all the models

All models use Cross-Entropy as a loss function. The initial learning rate was divided by five when the loss did not decrease by more than 10^{-4} for two consecutive epochs. The training stops after a maximum of 2000 epochs.

Table S5

Technical specifications to conduct the machine learning analyses.

Component	Version
Python	3.7.13
Scikit-learn	1.0.2
Numpy	1.21.6
SHAP	0.40.0

Classification of affective states on virtual agents: Method specifications

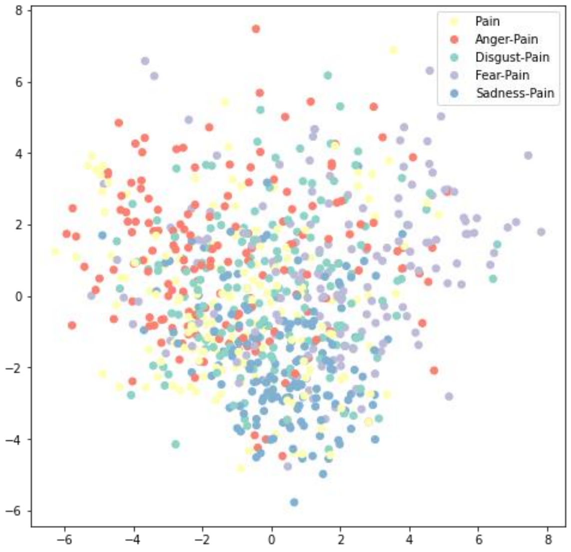

Figure S10. PCA on data from virtual agents, colored by labeled classes (affective states). It shows nontrivial linear separation between classes.

Table S6

Hyperparameters for the MLP affective states classification model on data from virtual agents.

Hyperparameter	Value
numpy.random.seed	75635
Number of units in the hidden layer	(130)
Activation function	'relu'
solver	'sgd'
learning_rate_init	0.001
max_iter	2000
learning_rate	'adaptive'
Other unmodified parameters	The default values of version 1.0.2 of scikit-learn

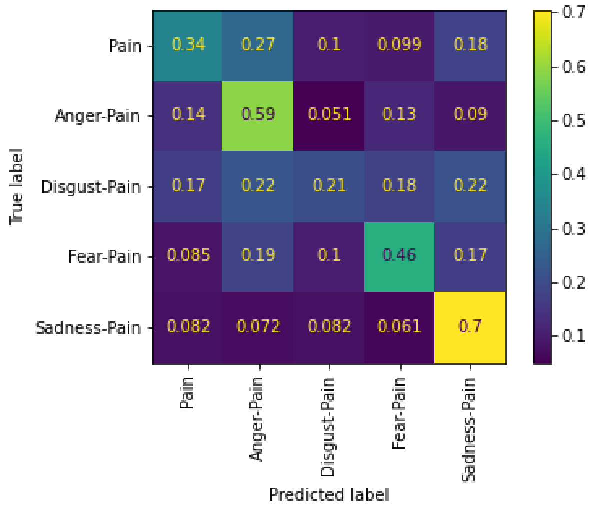

Figure S11. Normalized confusion matrix for the MLP affective states classification model on data from virtual agents.

Classification of affective states on participants' faces: Method specifications

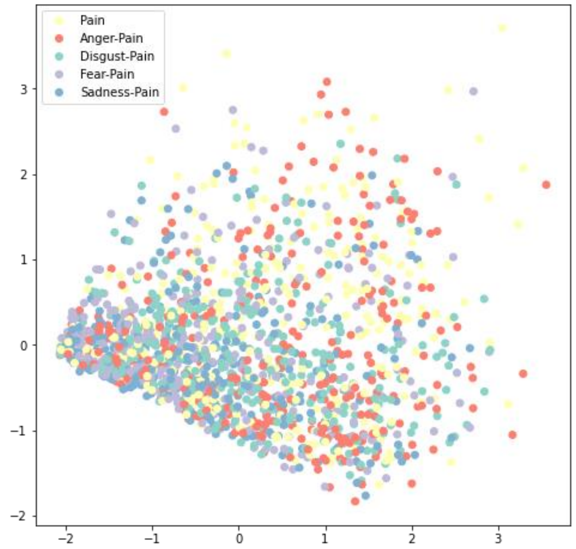

Figure S12. PCA on data from participants' faces, colored by labeled classes (affective states). It shows nontrivial linear separation between classes.

Table S7

Hyperparameters for the MLP affective states classification model on data from participants' faces.

Hyperparameter	Value
numpy.random.seed	75635
Number of units in the hidden layer	(120)
Activation function	'relu'
solver	'sgd'
learning_rate_init	0.01
max_iter	2000
learning_rate	'adaptive'
Other unmodified parameters	The default values of version 1.0.2 of scikit-learn

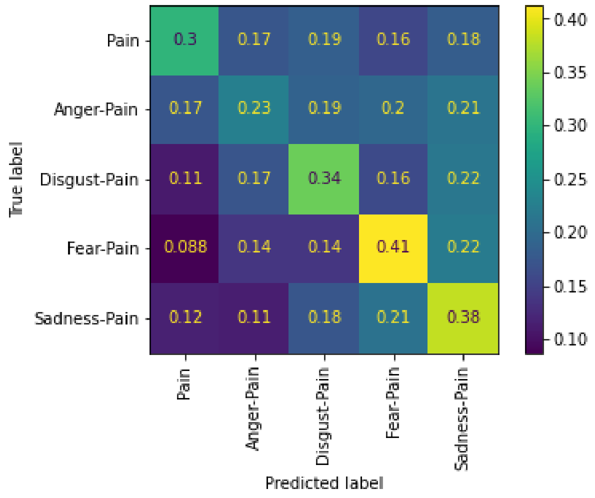

Figure S13. Normalized confusion matrix for the MLP affective state classification model on data from participants' faces.

Classification of imitated and posed facial configurations: Method specifications

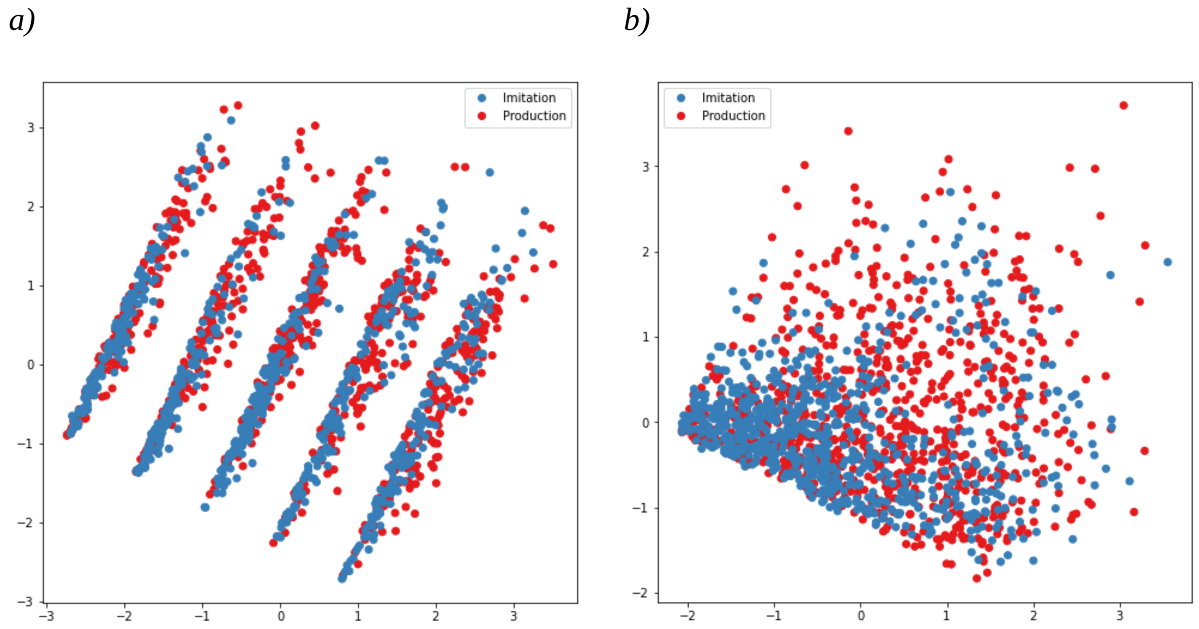

Figure S14. PCA on data from participants' faces, colored by labeled classes (imitated and posed facial configurations). It shows nontrivial linear separation between classes. *a)* Including the five affective state labels as a feature to create a visualization by affective state. *b)* All affective state labels pooled together.

Table S8

Hyperparameters for the MLP imitated or posed faces classification model.

Hyperparameter	Value
numpy.random.seed	75635
Number of units in the hidden layer	(50)
Activation function	'relu'
solver	'sgd'
learning_rate_init	0.001
max_iter	2000
learning_rate	'adaptive'
Other unmodified parameters	The default values of version 1.02 of scikit-learn

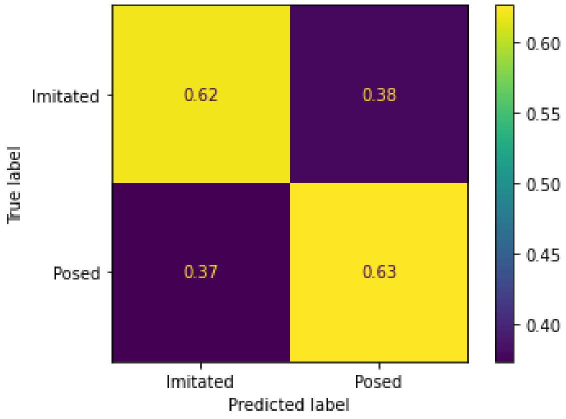

Figure S15. Normalized confusion matrix for the MLP imitated or posed faces classification model.

Appendix 6. Examples of Facial Configurations on Virtual Agents

a)

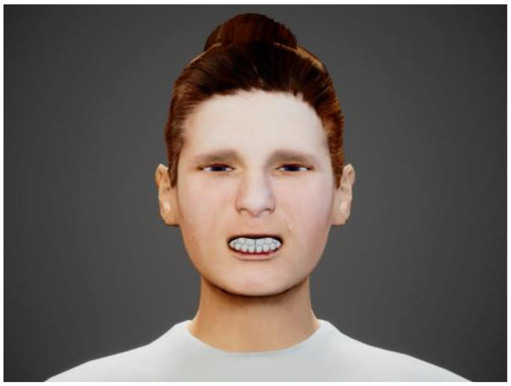

b)

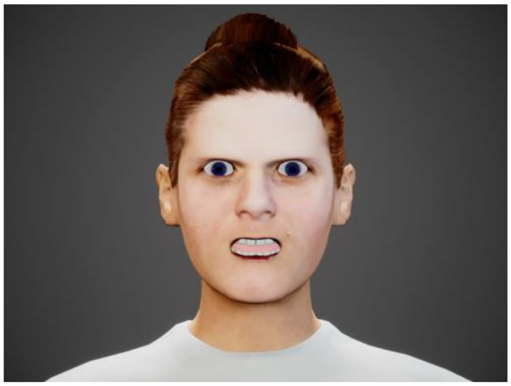

c)

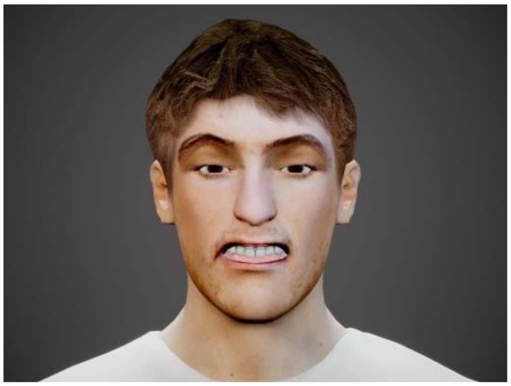

d)

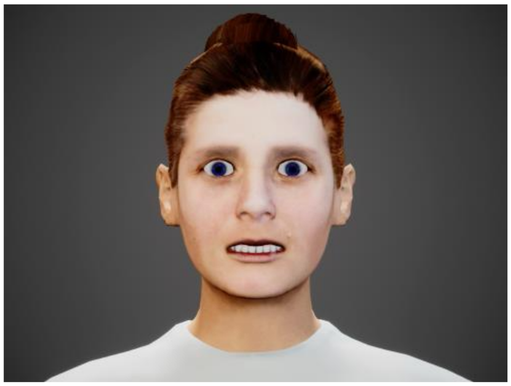

e)

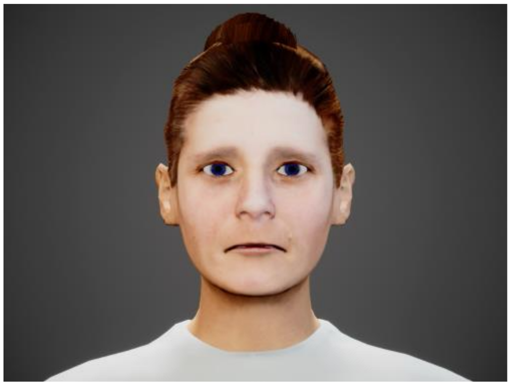

Figure S16. Examples of facial configurations on the virtual agents best classified by the machine learning model for the scenarios of: *a)* Pain; *b)* Anger-Pain; *c)* Disgust-Pain; *d)* Fear-Pain; and *e)* Sadness-Pain.

Appendix 7. Comparisons of the Absolute Means of SHAP between Action Units

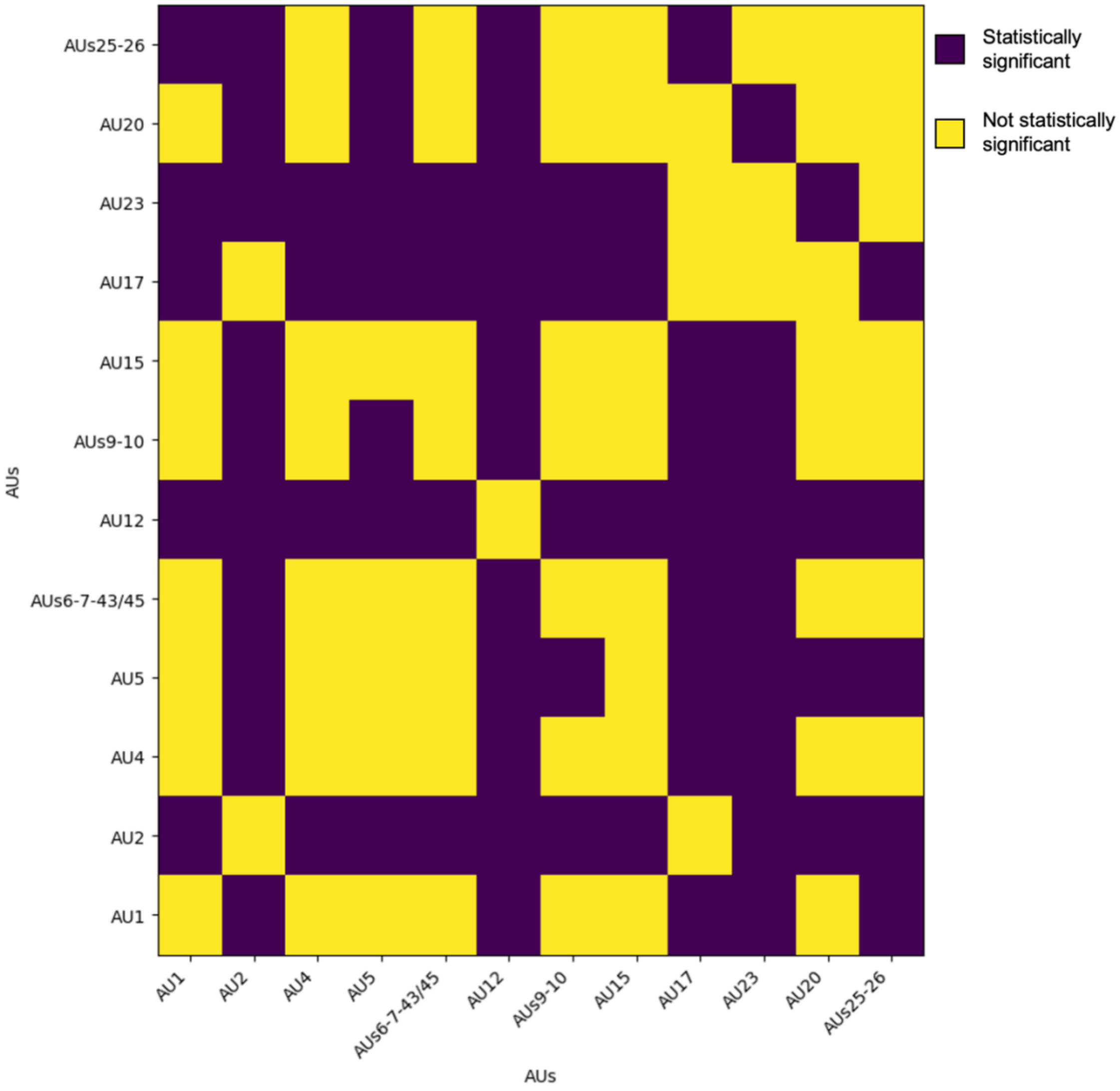

Figure S17. Visualization of the pairwise comparisons between the absolute SHAP means of Action Units (AUs) depicted on the virtual agents to predict affective states. Because of the skewed distribution of the SHAP, Wilcoxon signed-rank tests were used. The familywise inflation of Type I error rate from the multiple tests was controlled for by the Bonferroni adjustment ($\alpha_{post\ hoc}$ = .05/66 = .0008). Statistically significant comparisons are identified in purple, while the non-statistically significant ones are identified in yellow. The absolute SHAP mean of the top-rankest feature, AU 5, is not statistically significant from those of AU 1, AU 4, AUs 6-7-43/45, and AU 15. The absolute SHAP mean of the lowest-ranked feature, AU 12, is statistically lower than those of all higher-ranked features.

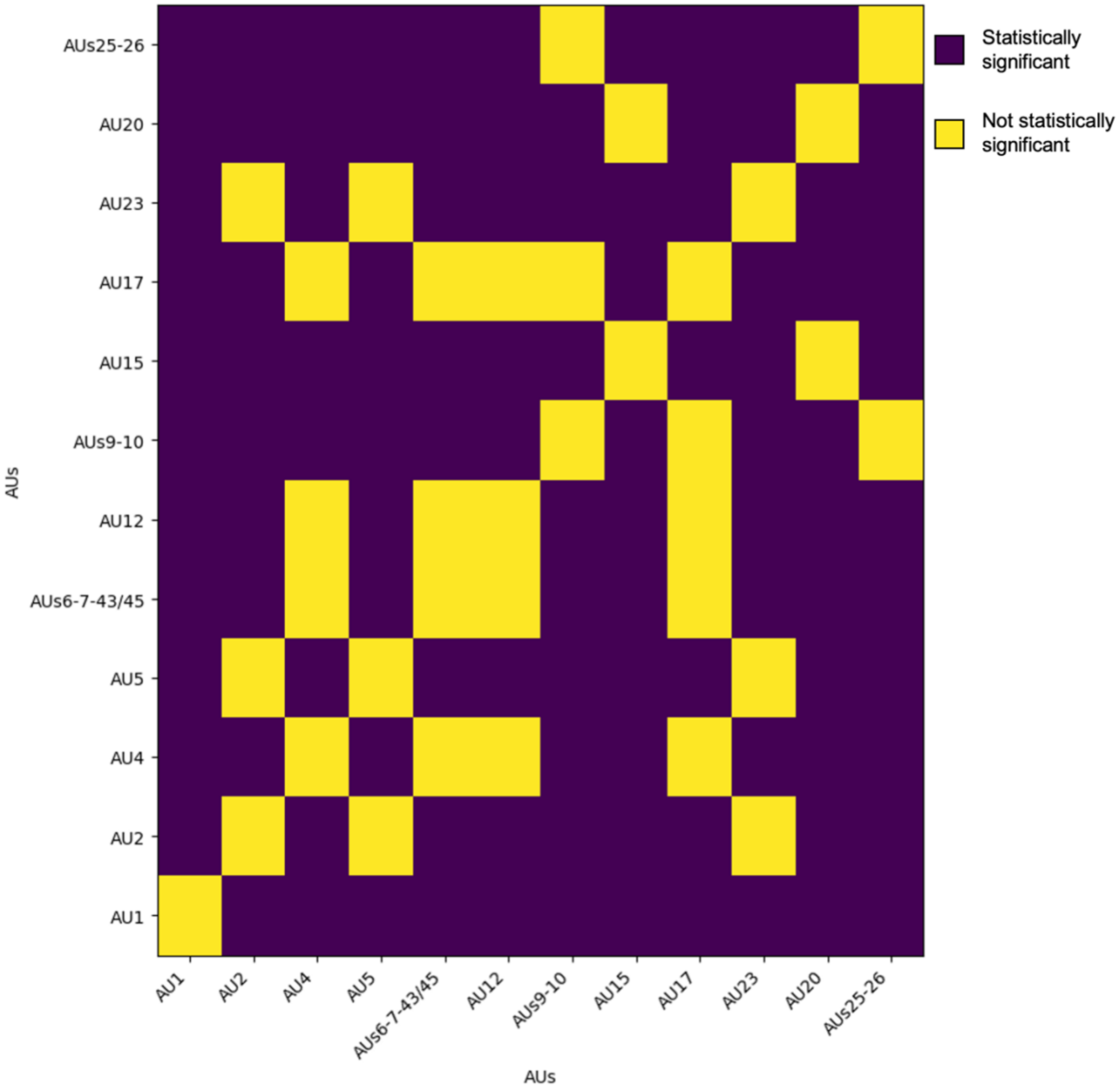

Figure S18. Visualization of the pairwise comparisons between the absolute SHAP means of Action Units (AUs) measured on the participants' faces to predict affective states. Because of the skewed distribution of the SHAP, Wilcoxon signed-rank tests were used. The familywise inflation of Type I error rate from the multiple tests was controlled for by the Bonferroni adjustment ($\alpha_{post\ hoc}$ = .05/66 = .0008). Statistically significant comparisons are identified in purple, while the non-statistically significant ones are identified in yellow. The absolute SHAP mean of the top-rankest feature, AU 4, is not statistically significant from those of AUs 6-7-43/45, AU 12, and AU 17. The absolute SHAP mean of the lowest-ranked feature, AU 2, is not statistically significant from those of AUs 5 and 23.

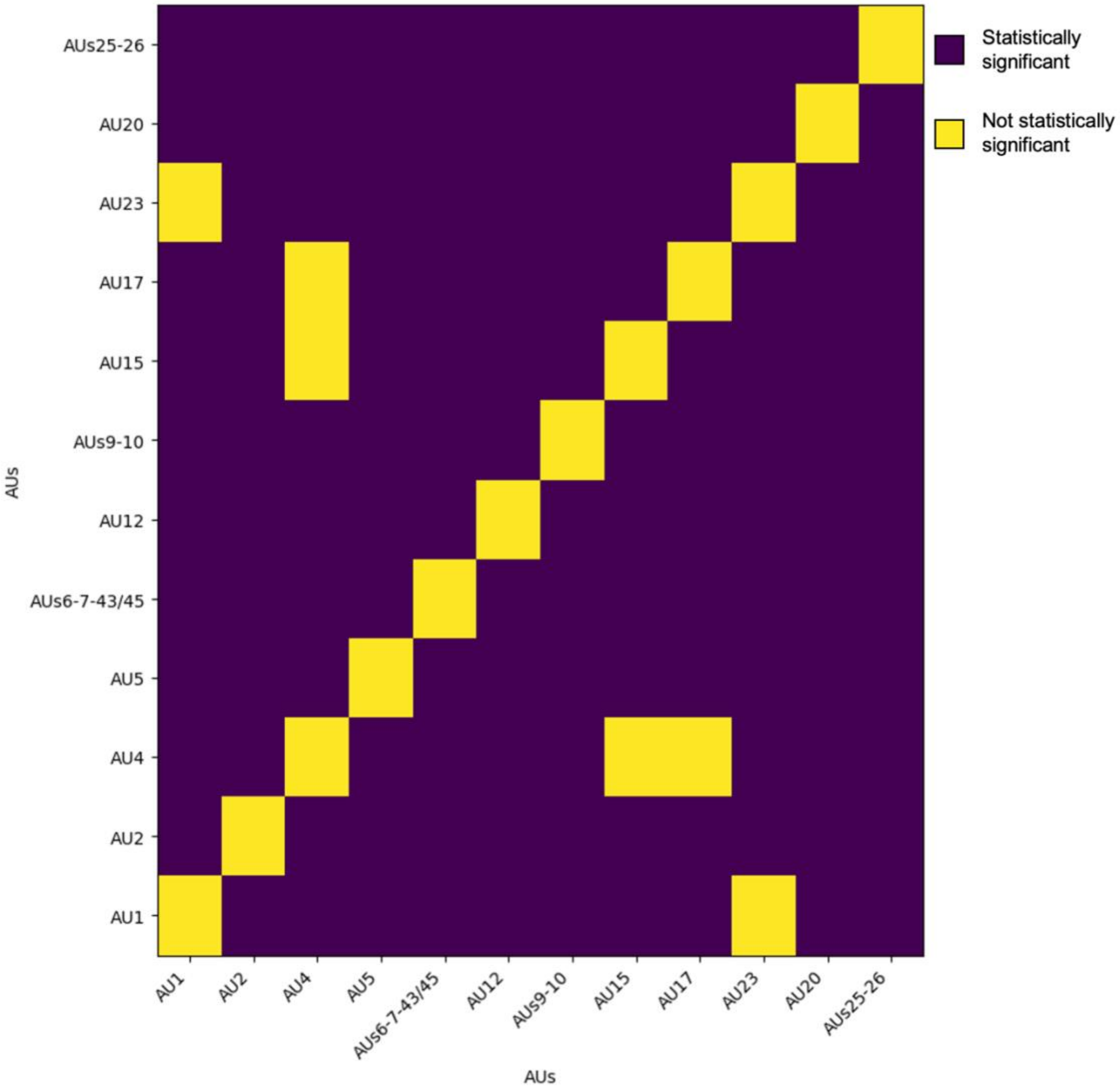

Figure S19. Visualization of the pairwise comparisons between the absolute SHAP means of Action Units (AUs) measured on the participants' faces to predict the type of facial configurations. Because of the skewed distribution of the SHAP, Wilcoxon signed-rank tests were used. The familywise inflation of Type I error rate from the multiple tests was controlled for by the Bonferroni adjustment ($\alpha_{post\ hoc}$ = .05/66 = .0008). Statistically significant comparisons are identified in purple, while the non-statistically significant ones are identified in yellow. The absolute SHAP mean of the top-ranked feature, AU 12, is statistically higher than those of all lower-ranked features. The absolute SHAP mean of the lowest-ranked feature, AU 2, is statistically lower than those of all higher-ranked features.

Appendix 8. Exploratory Analyses - Level of Affective Information in Both Representations

As a further exploratory step towards examining the facial configurations of combined states, the affective information expressed on virtual agents and participants' faces were compared for each affective state (Pain, Anger-Pain, Disgust-Pain, Fear-Pain, and Sadness-Pain). This new analysis provides a synthesis of the results on AUs and it can be used to link back to the combined states induced by the scenarios. An index for each negative emotion combined with pain (Anger, Disgust, Fear, and Sadness) was calculated. Each index was computed as the average of AUs most associated with the negative emotions (see Table 1 in the main text) and found important in the SHAP analyses either on VAs (see Figure 2 in the main text) or participants' faces (see Figure 4 in the main text). For the Anger index, the AUs that were averaged were 4, 5, 6-7-43/45, 9-10, and 23. For the Disgust index, the AUs that were averaged were 9-10, and 17. For the Fear index, the AUs that were averaged were 1, 2, 4, 5, 20, and 25-26. For the Sadness index, the AUs that were averaged were 1, 4, 15, and 17. The Pain index was also included (see the main text). Each index resulted in a scale ranging from 0 to 1. Five LMMs were conducted for the values of indexes to compare the level of the different affective states expressed on the VAs and the participants' faces for each type of scenarios (combined affective states). Two within-subjects variables were included as fixed effects factors: *Affective indexes* (5 conditions: Pain, Anger, Disgust, Fear, and Sadness) and *Type of facial representation* (2 conditions: VAs and participants' faces). The familywise inflation of Type I error rate from the multiple LMMs was controlled for by the Bonferroni adjustment ($\alpha_{univariate} = .05/5 = .01$). Figure S20 presents the results of all LMMs.

Two warnings are in order when interpreting these results. Firstly, some AUs were most intensely represented on the VAs and the participants' faces (e.g., AU 4) and could

disproportionately influence the value of the indexes. Secondly, based on the objective of this study (i.e., to examine the interaction between pain and negative emotions), some AUs were coupled in the analyses and the design of the interactive VAs application based on pain literature (i.e., AUs 6-7-43/45, 9-10, and 25-26). However, AUs most associated with some emotions (see Table 1 in the main text) sometimes include only one from these clusters of AUs (e.g., AU 7 for anger). Consequently, the results of these analyses must be interpreted cautiously in light of the results for each AU or cluster of AUs, and the pain information that can be derived from them (see *Results* in the main text).

The LMM on the Pain scenarios showed a statistically significant interaction effect of *Affective indexes* X *Type of facial representation* ($F(4, 243) = 3.77$, $p = .005$, $\eta^2_p = .06$). All indexes were higher on the VAs than participants' faces (ps < .001). On the VAs ($F(4, 243) = 18.75$, $p < .001$, $\eta^2_p = .24$) and the participants' faces ($F(4, 243) = 9.09$, $p < .001$, $\eta^2_p = .13$), the Disgust index was lower than the Pain index (ps ≤ .001) and the Anger index (ps ≤ .003). On the VAs, the Disgust index was also lower than the Fear index as well as the Sadness index (ps < .001). On the participants' faces, the Pain index was higher than the Sadness and Fear indexes (ps ≤ .001).

The LMM on the Anger-Pain scenarios showed a statistically significant main effect of *Affective indexes* ($F(4, 243) = 13.04$, $p < .001$, $\eta^2_p = .18$) and a statistically significant main effect of *Type of facial representation* ($F(1, 243) = 518.72$, $p < .001$, $\eta^2_p = .68$). No interaction effect was found statistically significant ($F(4, 243) = 1.32$, $p = .264$, $\eta^2_p = .02$). All indexes were higher on the VAs than participants' faces. The affective index associated with the highest value was Pain. ($ps_{bonf} \leq .007$). The Anger index was also higher than the Disgust index ($p_{bonf} = .004$).

The LMM on the Disgust-Pain scenarios showed a statistically significant interaction effect of *Affective indexes* X *Type of facial representation* ($F(4, 243) = 8.63$, $p < .001$, $\eta^2_p = .12$). All indexes were higher on the VAs than participants' faces (*p*s < .001). On the VAs ($F(4, 243) = 16.80$, $p < .001$, $\eta^2_p = .22$) and the participants' faces ($F(4, 243) = 8.45$, $p < .001$, $\eta^2_p = .12$), the Disgust index was lower than the Pain index (*p*s < .001). On the VAs, the Disgust index was also lower than the Sadness index as well as the Fear index (*p*s < .001) and the Anger index was lower than the Sadness, Fear, and Pain indexes (*p*s ≤ .001). On the participants' faces, the Pain index was higher than the Fear index ($p < .001$) and the Disgust index was lower than the Anger index ($p = .004$).

The LMM on the Fear-Pain scenarios showed a statistically significant interaction effect of *Affective indexes* X *Type of facial representation* ($F(4, 238.01) = 21.06$, $p < .001$, $\eta^2_p = .26$). All indexes were higher on the VAs than participants' faces (*p*s < .001). On the VAs ($F(4, 238.01) = 43.19$, $p < .001$, $\eta^2_p = .42$), the affective index associated with the highest value was Fear (*p*s < .001). The Sadness index was also higher than the Pain, Anger, and Disgust indexes (*p*s < .001), and the Disgust index was lower than the Pain and Anger indexes (*p*s ≤ .001). On the participants' faces ($F(4, 238.01) = 4.93$, $p = .001$, $\eta^2_p = .08$), the Pain index was higher than those of Fear ($p = .004$) and Disgust ($p < .001$).

The LMM on the Sadness-Pain scenarios showed a statistically significant interaction effect of *Affective indexes* X *Type of facial representation* ($F(4, 243) = 36.37$, $p < .001$, $\eta^2_p = .37$). All indexes were higher on the VAs than participants' faces (*p*s < .001). On the VAs ($F(4, 243) = 86.17$, $p < .001$, $\eta^2_p = .59$), the affective index associated with the highest value was Sadness (*p*s < .001). The Fear index was also higher than that of Anger and Disgust (*p*s < .001), and the Anger index was higher than the Disgust index ($p < .001$). On the participants' faces

($F(4, 243) = 5.85$, $p < .001$, $\eta^2_p = .09$), the Pain index was higher than the Fear and Disgust indexes (*p*s < .001). The Sadness index was also higher than the Disgust index ($p = .004$).

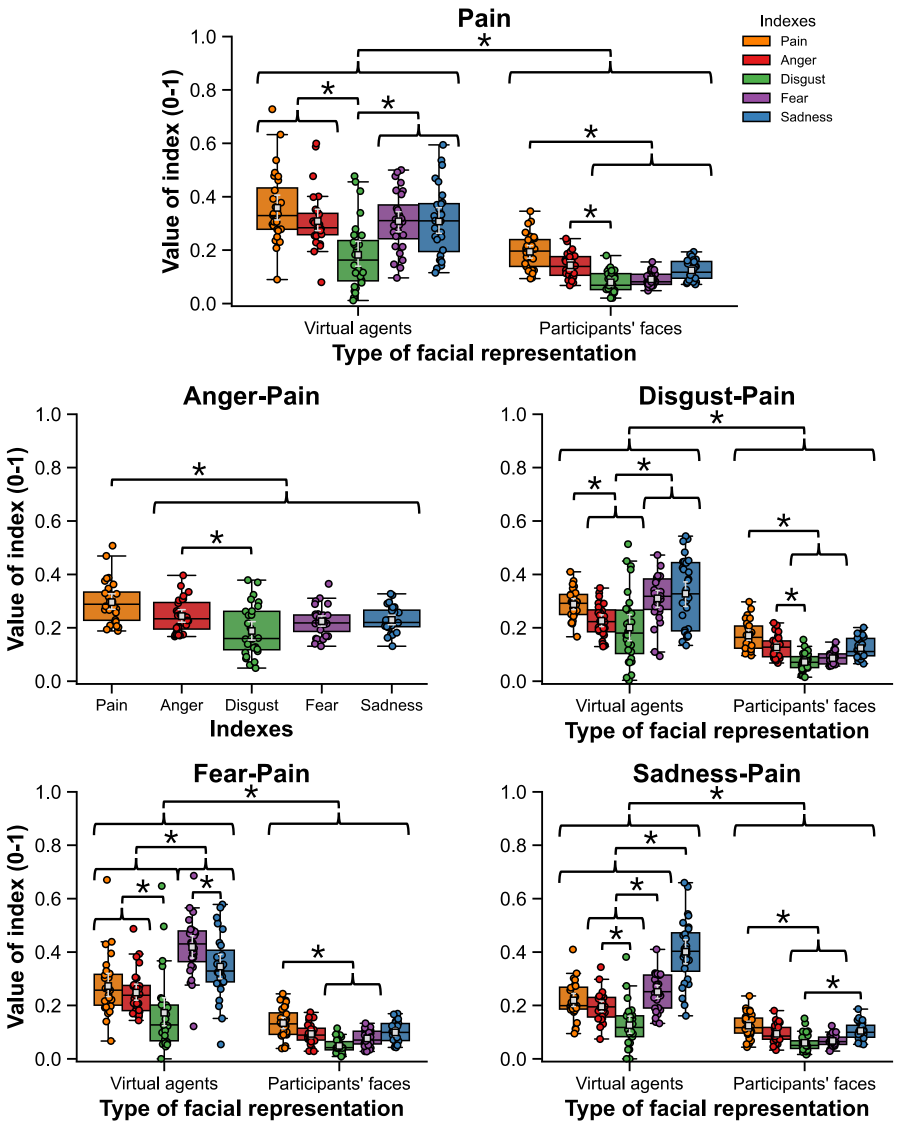

Figure S20. Results per affective states (Pain, Anger-Pain, Disgust-Pain, Fear-Pain, and Sadness-Pain) of the interaction effect of Affective indexes and Type of facial representation (or main effect of Affective Indexes for Anger-Pain) on the mean intensity value of indexes. The colored box-and-whisker plots and the colored points show the data distribution ($n = 28$ and $n = 27$ for Fear-Pain) for each affective index on the virtual agents and the participants' faces (averaged for Anger-Pain). The whiskers present the minimum and maximum values, the vertical length of the box presents the interquartile range, and the horizontal line within the box presents the median. The grey squares show the mean scores for each affective state on the virtual agents and the participants' faces, and grey error bars indicate a 95% CI. * $ps_{\text{bonf}} < .05$

Supplementary References

1. Mori, M., MacDorman, K. F. & Kageki, N. The Uncanny Valley. *IEEE Robot. Autom. Mag.* **19**, 98–100 (2012).
2. Groom, V. *et al.* Evaluating the effects of behavioral realism in embodied agents. *Int. J. Hum. Comput. Stud.* **67**, 842–849 (2009).
